# Supplementary material for: Bone morphogenetic protein (BMP) signaling determines neuroblastoma cell fate and sensitivity to retinoic acid
Source: Nat Commun. 2025 Feb 28;16:2036. doi: 10.1038/s41467-025-57185-y (PMC11871043; doi:10.1038/s41467-025-57185-y)
Supplement: Supplementary file 1 — Supplementary Information [file 41467_2025_57185_MOESM1_ESM.pdf]

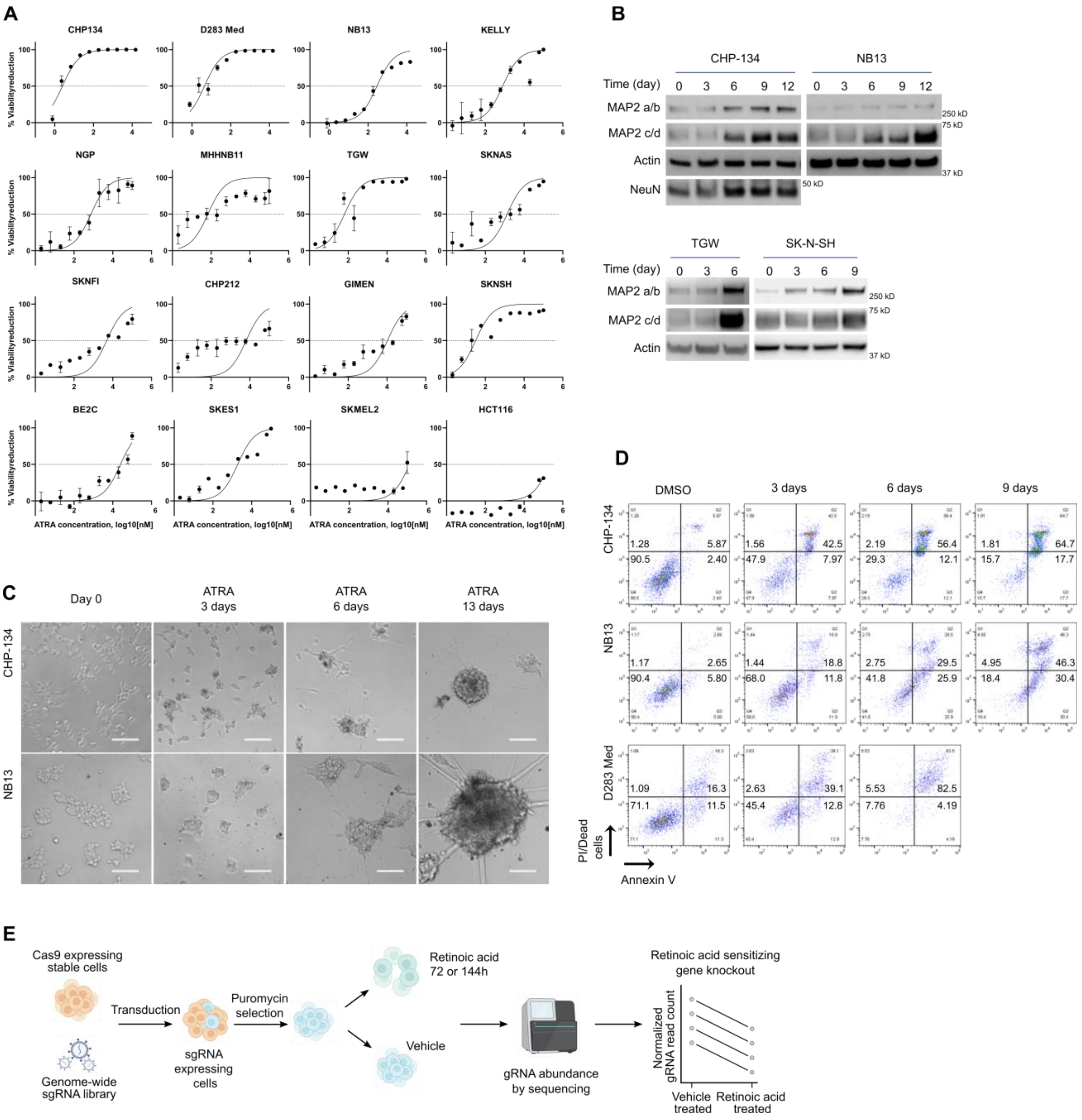

A
CHP134
D283 Med
NB13
KELLY
NGP
MHHNB11
TGW
SKNAS
SKNFI
CHP212
GIMEN
SKNSH
BE2C
SKES1
SKMEL2
HCT116
% Viabilityreduction
ATRA concentration, log10[nM]
B
CHP-134
NB13
Time (day)
0 3 6 9 12
MAP2 a/b
MAP2 c/d
Actin
NeuN
250 kD
75 kD
37 kD
50 kD
TGW
SK-N-SH
C
Day 0
ATRA 3 days
ATRA 6 days
ATRA 13 days
CHP-134
NB13
D
DMSO
3 days
6 days
9 days
PI/Dead cells
Annexin V
E
Cas9 expressing stable cells
Transduction
Genome-wide sgRNA library
sgRNA expressing cells
Puromycin selection
Retinoic acid 72 or 144h
Vehicle
gRNA abundance by sequencing
Retinoic acid sensitizing gene knockout
Normalized gRNA read count
Vehicle treated
Retinoic acid treated

F

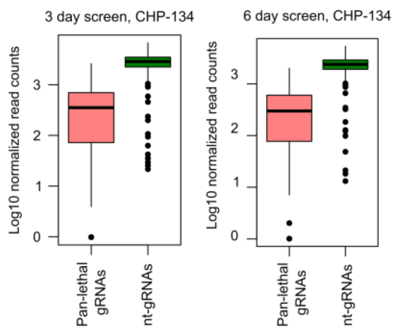

G

Index	Name	P-value	Adjusted p-value	Odds Ratio	Combined score
1	ALK2 pathway	1.04E-06	0.000149	43.35	597.18
2	BMP signaling and regulation	1.76E-06	0.000205	37.16	492.41
3	Signaling by bone morphogenetic protein (BMP)	1.65E-07	5.44E-05	22.87	357.11
4	BMP signaling pathway in stem cells	6.35E-06	0.000495	26.01	311.2
5	p27 phosphorylation regulation during cell cycle progression	8.17E-05	0.002604	23.06	217.01
6	G2 phase pathway	0.003502	0.03458	34.41	194.58
7	TGF-beta signaling in development	7.78E-06	0.000545	15.64	183.96
8	Cell cycle: G1/S checkpoint	1.23E-05	0.000617	14.21	160.68
9	Rb tumor suppressor/checkpoint signaling in response to DNA damage	0.000199	0.004303	17.29	147.36
10	Transfer RNA aminoacylation	2.28E-06	0.000228	11.02	143.2

H

Depletion (negative selection)

Gene	negative rank	negative p-value
FKBP1A	1	2.59E-07
SMURF2	20	2.04E-04
SMAD7	171	4.99E-03
SMAD6	304	1.30E-02

Enrichment (positive selection)

Gene	positive rank	positive p-value
BMPR2	81	2.28E-03
RARA	91	2.76E-03
ACVR1	178	6.62E-03
SMAD4	245	0.01

P = 2.9 × 10^{-15} from Fisher's exact test for 3-day and 6-day screens.

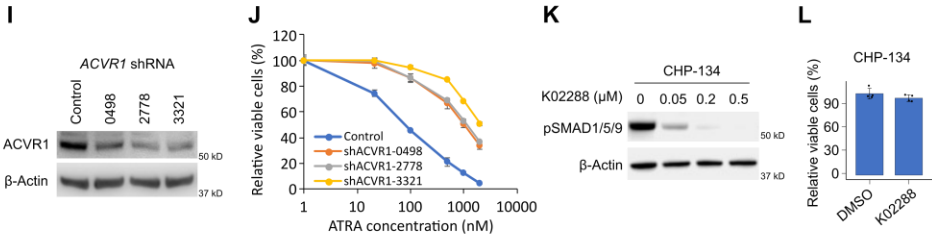

Supplementary Figure 1

A. Dose response curve of ATRA in 12 neuroblastoma cell lines and 4 non-neuroblastoma cell lines. The data were retrieved from Lee et al (PMID 37957169) (Lee, Wright et al. 2023), except CHP-134, D283 Med, and NB13. The curve fit was performed using GraphPad Prism 9 (nonlinear regression, log(agonist) vs. normalized response). Data represent the mean ± SEM (n = 3).
B. Western blots showing expression of differentiation markers in CHP-134, NB13, TGW, and SK-N-SH cell lines treated with 0.5 µM (CHP-134) or 2µM (NB13, TGW, SK-N-SH) of ATRA for the time indicated.
C. Images showing neurite growth in CHP-134 and NB13 cell lines after RA treatment (0.5 µM for CHP-134 and 2 µM for NB13). Day 0 indicates DMSO treatment (< 0.01% of DMSO, the same concentration as in RA treated cells) for 13 days. Scale bar = 100 µm. Representative results of 3 independent replicates are shown.
D. Flow cytometry results showing apoptosis in CHP-134, NB13, and D283 Med cell lines treated with RA (2µM). Apoptotic cells were stained positive with annexin V and dead cells were stained positive with PI. Representative results of three independent replicates are shown.
E. Schematic of CRISPR knockout screening strategy. BioRender was used to generate the schematic (Created in BioRender. Pan, M. (2025) https://BioRender.com/n24x642).
F. Read counts of 55 pan-essential genes from DepMap and 1,000 non-targeting negative control gRNAs used in the genome-wide screens.
G. Table of the top pathways identified from the genome-wide CRISPR knockout screen in CHP-134 cell line, as reported by Enrichr (https://maayanlab.cloud/Enrichr/). Top 1% of genes with lowest positive RRA score and top 1% of genes with lowest negative RRA score (191 + 191 genes) were used as input.

H. CRISPR knockout screen in CHP-134 cell line treated with 2 µM ATRA for 3 days. BMP-related genes and RARA are shown. Genes are ranked by RRA score (rank #1 has the lowest RRA score).
I. Western blots showing ACVR1 expression in CHP-134 cells following lentiviral shRNA knockdown. Control is a control shRNA with no specific target in human genome; 0498, 2778, and 3321 are three independent shRNAs targeting ACVR1.
J. Cell viability in CHP-134 cells with ACVR1 knockdown. Viability was measured with MTS assay and normalized to DMSO treated cells. Data represent the mean ± SD of 9 independent replicates (n = 9).
K. Western blots showing expression of phosphorylated SMAD1/5/9 in CHP-134 cells treated with K02288 for 3 days.
L. Viability of DMSO and 0.5 µM K02288 treated CHP-134 cells. Cells were treated for 3 days. Data represent the mean ± SD of 4 independent replicates (n = 4).

For panel J and L, *P*-values are included in the Source Data file.. For panel B, I, and K, representative results of 3 independent replicates are shown, β-Actin was used as a loading control. Source data are provided as a Source Data file.

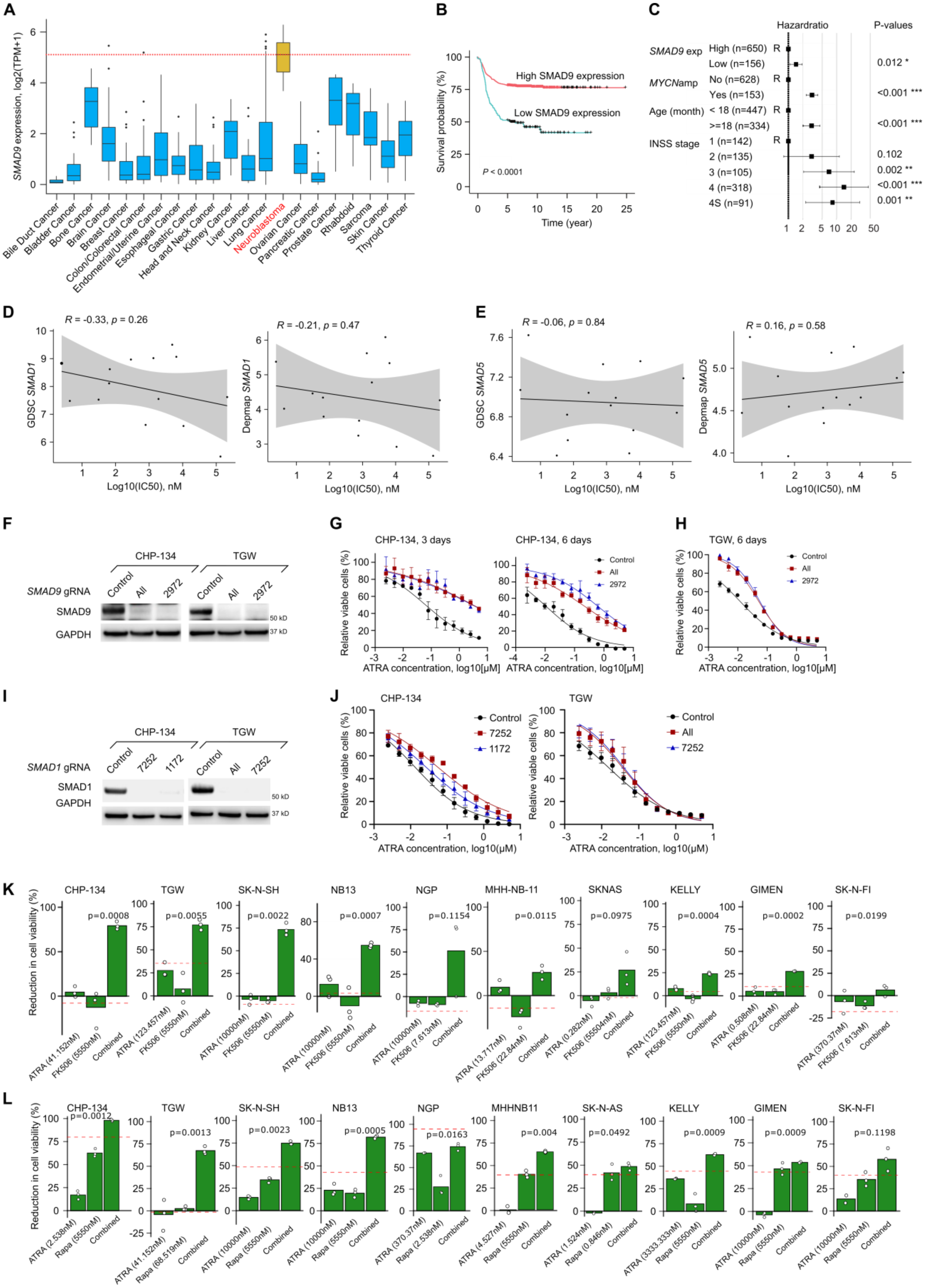

A
SMAD9 expression, log2(TPM+1)
Bile Duct Cancer
Bladder Cancer
Bone Cancer
Brain Cancer
Breast Cancer
Colon/Colorectal Cancer
Endometrial/Uterine Cancer
Esophageal Cancer
Gastric Cancer
Head and Neck Cancer
Kidney Cancer
Liver Cancer
Lung Cancer
Neuroblastoma
Ovarian Cancer
Pancreatic Cancer
Prostate Cancer
Rhabdoid
Sarcoma
Skin Cancer
Thyroid Cancer
B
Survival probability (%)
High SMAD9 expression
Low SMAD9 expression
P < 0.0001
Time (year)
C
Hazardratio
P-values
SMAD9 exp
High (n=650) R
Low (n=156)
0.012 *
MYCNamp
No (n=628) R
Yes (n=153)
<0.001 ***
Age (month)
< 18 (n=447) R
>=18 (n=334)
<0.001 ***
INSS stage
1 (n=142) R
2 (n=135)
0.102
3 (n=105)
0.002 **
4 (n=318)
<0.001 ***
4S (n=91)
0.001 **
D
R = -0.33, p = 0.26
GDSC SMAD1
R = -0.21, p = 0.47
Depmap SMAD1
Log10(IC50), nM
E
R = -0.06, p = 0.84
GDSC SMAD5
R = 0.16, p = 0.58
Depmap SMAD5
Log10(IC50), nM
F
CHP-134
TGW
SMAD9 gRNA
Control
All
2972
SMAD9
GAPDH
50 kD
37 kD
G
CHP-134, 3 days
CHP-134, 6 days
Relative viable cells (%)
Control
All
2972
ATRA concentration, log10[μM]
H
TGW, 6 days
Relative viable cells (%)
Control
All
2972
ATRA concentration, log10[μM]
I
CHP-134
TGW
SMAD1 gRNA
Control
7252
1172
All
SMAD1
GAPDH
50 kD
37 kD
J
CHP-134
TGW
Relative viable cells (%)
Control
7252
1172
All
ATRA concentration, log10(μM)
K
Reduction in cell viability (%)
CHP-134 p=0.0008
ATRA (41.152nM)
FK506 (5550nM)
Combined
TGW p=0.0055
ATRA (123.457nM)
FK506 (5550nM)
Combined
SK-N-SH p=0.0022
ATRA (10000nM)
FK506 (5550nM)
Combined
NB13 p=0.0007
ATRA (10000nM)
FK506 (5550nM)
Combined
NGP p=0.1154
ATRA (10000nM)
FK506 (7.613nM)
Combined
MHH-NB-11 p=0.0115
ATRA (13.717nM)
FK506 (22.84nM)
Combined
SKNAS p=0.0975
ATRA (0.282nM)
FK506 (55504nM)
Combined
KELLY p=0.0004
ATRA (123.457nM)
FK506 (5550nM)
Combined
GIMEN p=0.0002
ATRA (0.508nM)
FK506 (22.84nM)
Combined
SK-N-FI p=0.0199
ATRA (370.37nM)
FK506 (7.613nM)
Combined
L
Reduction in cell viability (%)
CHP-134 p=0.0012
ATRA (2.538nM)
Rapa (5550nM)
Combined
TGW p=0.0013
ATRA (41.152nM)
Rapa (68.519nM)
Combined
SK-N-SH p=0.0023
ATRA (10000nM)
Rapa (5550nM)
Combined
NB13 p=0.0005
ATRA (10000nM)
Rapa (5550nM)
Combined
NGP p=0.0163
ATRA (370.37nM)
Rapa (2.538nM)
Combined
MHHNB11 p=0.004
ATRA (4.527nM)
Rapa (5550nM)
Combined
SK-N-AS p=0.0492
ATRA (1.524nM)
Rapa (0.846nM)
Combined
KELLY p=0.0009
ATRA (3333.333nM)
Rapa (5550nM)
Combined
GIMEN p=0.0009
ATRA (10000nM)
Rapa (5550nM)
Combined
SK-N-FI p=0.1198
ATRA (10000nM)
Rapa (5550nM)
Combined

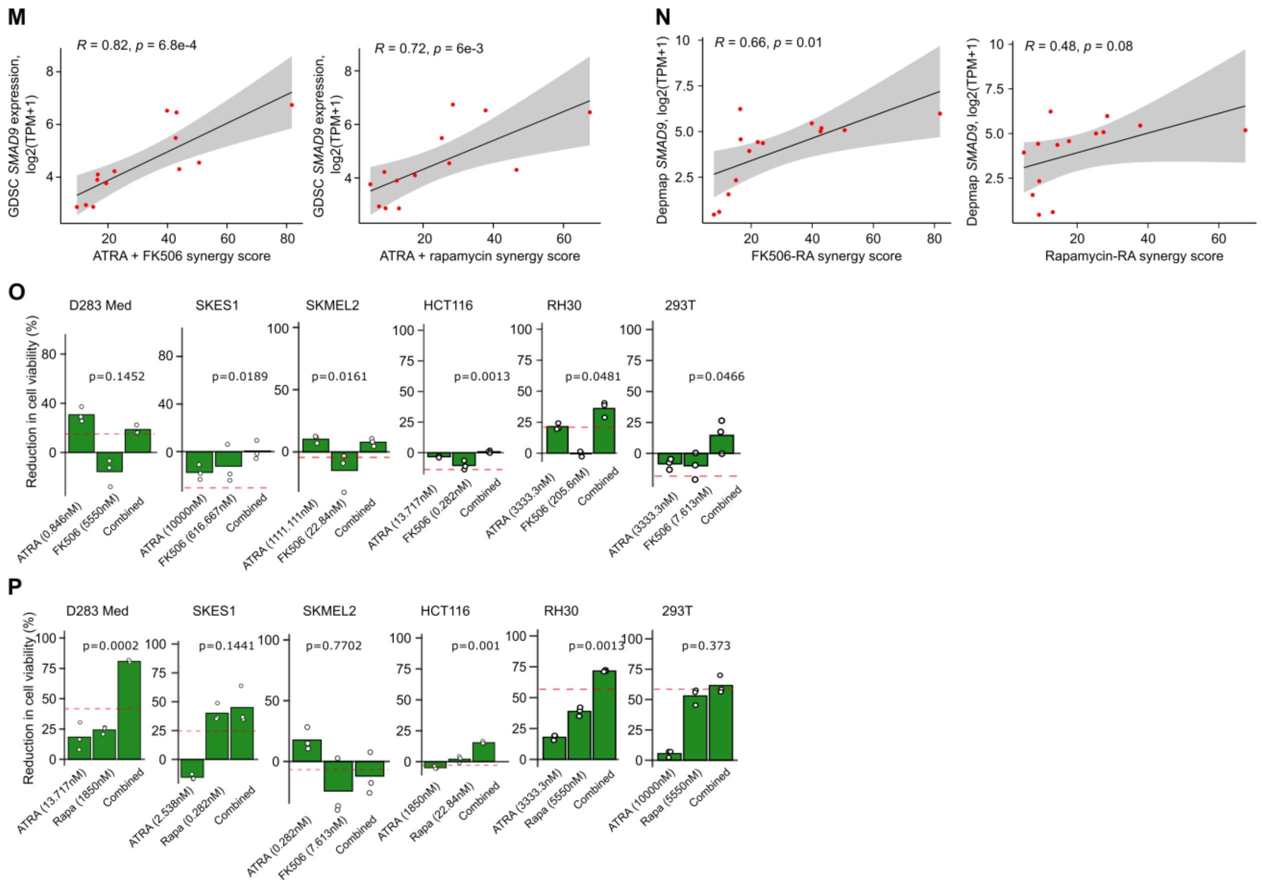

Supplementary Figure 2

A. *SMAD9* expression in all non-hematological cancer cell lines in the data from DepMap Expression Public 22Q4 (https://depmap.org/portal/gene/SMAD9?tab=characterization). Cancer types with ≤ 3 cell lines are not included. n = 759 cell lines in total.
B. Kaplan-Meier curves showing overall survival in Cangelosi *et al.* (2020), grouped by *SMAD9* expression. For plotting purposes samples in the bottom 20% are designated as low expression. *n* = 625 and *n* = 156 samples in the low and high groups respectively. The "+" indicates censoring events. *P*-values calculated using a Cox proportional hazards model.
C. Forest plot displaying the hazard ratio for *SMAD9* expression, along with other factors such as *MYCN* status, age, and INSS tumor stage. These hazard ratios were derived from a multivariate Cox proportional hazard model that jointly considers the association between overall survival and each of these factors.
D. Scatter plot of *SMAD1* RNA expression from GDSC (left) and Depmap (right) plotted against RA IC50 values. P-values were calculated with two-sided Pearson correlation tests.
E. Same as (A) but showing *SMAD5* expression and IC50.
F. Western blots showing SMAD9 protein levels in CHP-134 and TGW cell lines following CRISPR knockout. Control, negative control gRNA with no target in human genome; All, a mixture of 3 independent gRNAs targeting *SMAD9*; 2972, a single gRNA targeting *SMAD9*. GAPDH was used as loading control. Representative results of 3 independent replicates are shown.

G. Viability of CHP-134 cells following *SMAD9* knockout under treatment with RA for 3 days (left) or 6 days (right). Data represent the mean ± SD of 3 independent replicates (n = 3).
H. Viability of TGW cells following SMAD9 knockout under treatment with RA for 6 days. Data represent the mean ± SD of 3 independent replicates (n = 3).
I. Western blots showing SMAD1 protein levels in CHP-134 and TGW cell lines following CRISPR knockout. Control, negative control gRNA with no target in human genome; All, a mixture of 3 independent gRNAs targeting *SMAD1*; 7252 and 1172 are single gRNAs targeting *SMAD1*. GAPDH was used as a loading control. Representative results of 3 independent replicates are shown.
J. Viability of CHP-134 (left) and TGW (right) cells following *SMAD1* knockout under treatment with RA for 6 days. Data represent the mean ± SD of 6 independent replicates (n = 6).
K. Neuroblastoma cell lines were screened with combination of ATRA and FK506 as shown in Figure 2F-G. Bar plots of RA and FK506 combinations that conferred the maximum synergy scores from synergy calculations are shown here. White dots represent three independent experiments corresponding to score maxima. Red lines represent the expected result based on additivity alone. P-values were calculated from one-sample t-tests between the expected result and the observed cell response values from the combinations.
L. Same as (K) but showing RA and rapamycin combinations.
M. Scatter plot of SMAD9 RNA expression from GDSC plotted against highest synergy score of ATRA + FK506 combination (left) and RA + rapamycin combination (right) in the cell lines screened in our drug combination study shown in Fig. E. P-values were calculated with two-sided Pearson correlation tests.
N. Same as (M) but showing *SMAD9* expression from Depmap.
O. Same as (K) but in non-neuroblastoma cell lines.
P. Same as (K) but showing RA and rapamycin combinations in non-neuroblastoma cell lines.

For panel G, H, and J, *P*-values are included in the Source Data file. Source data are provided as a Source Data file.

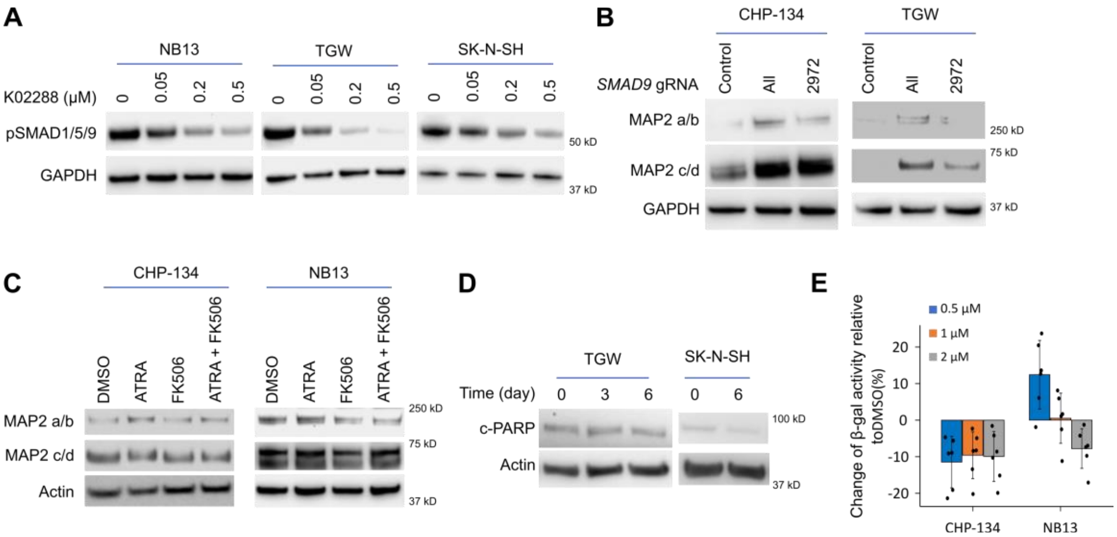

Supplementary Figure 3

A. Western blots showing BMP inhibitor K02288 suppressed BMP signaling activity (pSMAD1/5/9 level) in NB13, TGW, and SK-N-SH cell lines. Cells were treated with K02288 with indicated concentrations for 3 days.
B. Western blots for MAP2 expression in CHP-134 and TGW cell lines after SMAD9 CRISPR knockout. Control, negative control gRNA with no target in human genome; All, a mixture of 3 independent gRNAs targeting SMAD9; 2972, a single gRNA targeting SMAD9.
C. Western blots for MAP2 expression in CHP-134 and NB13 cell lines treated with indicated compounds for 3 days (2 µM RA and 0.1 µM FK506 for CHP-134, 0.05 µM RA and 5 µM FK506 for NB13).
D. Western blots for c-PARP expression in TGW and SK-N-SH cell lines treated with RA (2 µM for TGW and 5 µM for SK-N-SH) for the time indicated.
E. Senescence levels quantified by intensity of fluorescence in CHP-134 and NB13 cell lines treated with RA for 6 days. Data represent the mean ± SD of 6 independent replicates (n = 6). *P*-values are included in the Source Data file.

For panel A-D, β-Actin and GAPDH were used as loading control for western blotting. Representative results of 3 independent replicates are shown. Source data are provided as a Source Data file.

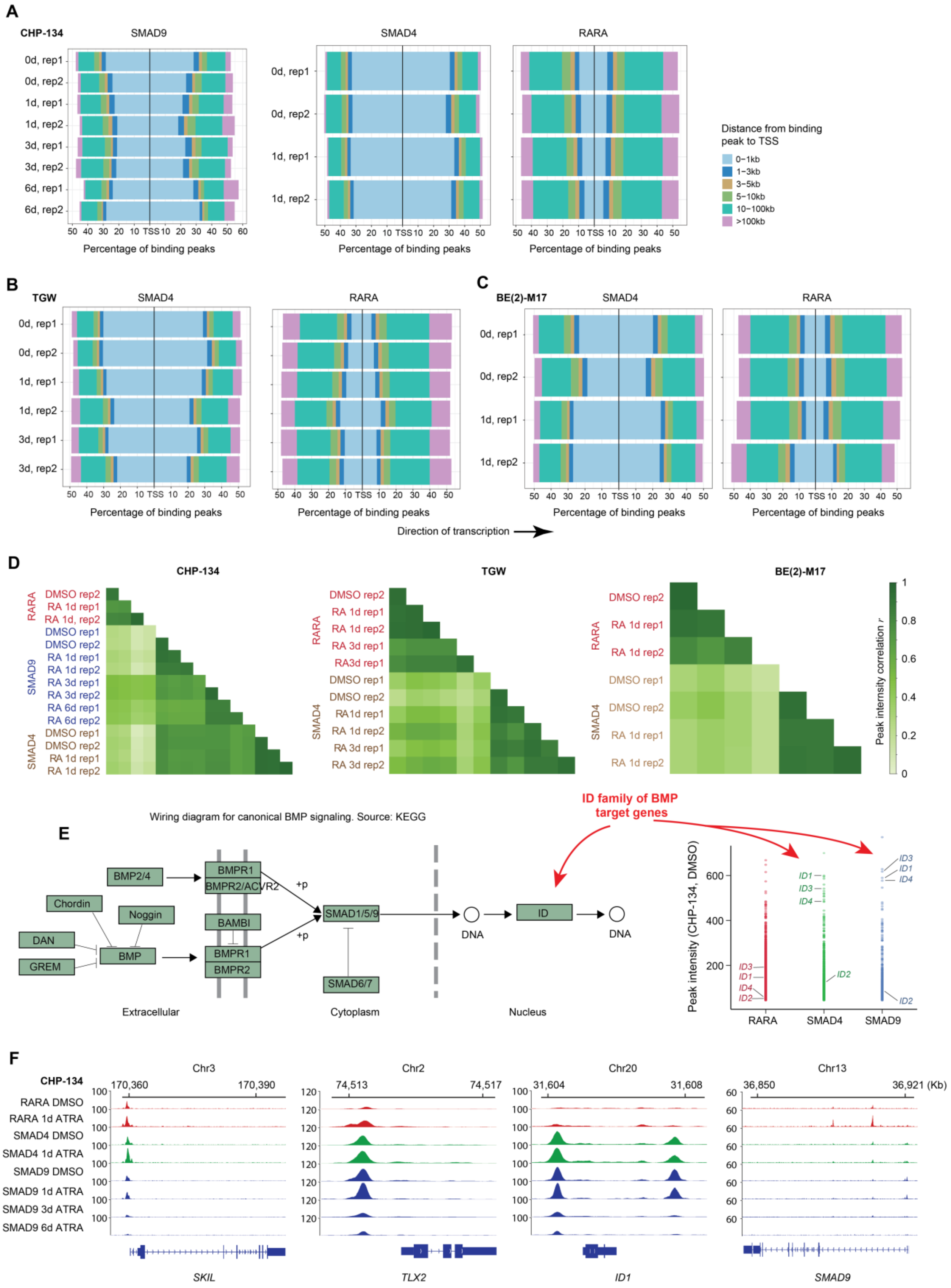
A
CHP-134
SMAD9
SMAD4
RARA
0d, rep1
0d, rep2
1d, rep1
1d, rep2
3d, rep1
3d, rep2
6d, rep1
6d, rep2
Percentage of binding peaks
Distance from binding peak to TSS
0−1kb
1−3kb
3−5kb
5−10kb
10−100kb
>100kb
B
TGW
C
BE(2)-M17
Direction of transcription
D
CHP-134
TGW
BE(2)-M17
DMSO rep2
RA 1d rep1
RA 1d, rep2
DMSO rep1
RA 1d rep2
RA 3d rep1
RA 3d rep2
RA 6d rep1
RA 6d rep2
Peak intensity correlation r
E
Wiring diagram for canonical BMP signaling. Source: KEGG
BMP2/4
Chordin
Noggin
DAN
GREM
BMP
BMPR1
BMPR2/ACVR2
BAMBI
BMPR2
SMAD1/5/9
SMAD6/7
+p
DNA
ID
Extracellular
Cytoplasm
Nucleus
ID family of BMP target genes
Peak intensity (CHP-134, DMSO)
ID1
ID2
ID3
ID4
F
CHP-134
Chr3
Chr2
Chr20
Chr13
170,360
170,390
74,513
74,517
31,604
31,608
36,850
36,921 (Kb)
RARA DMSO
RARA 1d ATRA
SMAD4 DMSO
SMAD4 1d ATRA
SMAD9 DMSO
SMAD9 1d ATRA
SMAD9 3d ATRA
SMAD9 6d ATRA
SKIL
TLX2
ID1
SMAD9

Supplementary Figure 4: Global summary/QC plots for the ChIP-seq data collected.

A. The percentage of binding peaks (x axis) at various distances from transcription start sites (TSS; colors). Data shown for SMAD9 (left), SMAD4 (middle), and RARA (right) ChIP-seq profiles in CHP-134. Each row represents a sample, with the sample name indicating the duration of 2 µM ATRA treatment (0, 1, 3, or 6 days) and the replicate number (rep 1 or 2) number. RARA bindings sites are more dispersed across the genome, compared to SMAD profiles.
B. Like A but for TGW. Cells were treated with DMSO or 2 µM ATRA for 1 day or 0.5 µM ATRA for 3 days.
C. Like A but for BE(2)-M17. Cells were treated with DMSO or 2 µM ATRA for 1 day.
D. Hierarchical clustering of Pearson’s correlations of shared-peak binding intensities between each pair of ChIP-seq samples for CHP-134 (left), TGW (middle) and BE(2)-M17 (right). The color indicates the factor profiled. In all cases, the strongest clustering is by technical replicate, followed by ChIP-mark and ATRA treatment, indicating the reproducibility/validity of our ChIP-seq data.
E. Stripchart (right panel) showing the genes bound by RARA, SMAD4, and SMAD9 in DMSO treated CHP-134 cells (x axis). The y axis indicates the peak binding intensity. As expected, the ID family of genes (the canonical targets of BMP signaling; left panel) are strongly bound by SMAD4 and SMAD9 in our data, indicating the data are reliable.
F. Selected genome browser tracks displaying the binding of RARA, SMAD4, and SMAD9 on four BMP target genes (*SKIL*, *TLX2*, *ID1*, and *SMAD9*) under DMSO or ATRA treatment in CHP-134, illustrating co-binding of these factors at many sites. SMAD9 binding is also lost over the treatment course, consistent with loss of SMAD9 mRNA expression in our RNA-seq data. Numbers on y-axis indicate the maximum normalized peak intensity.

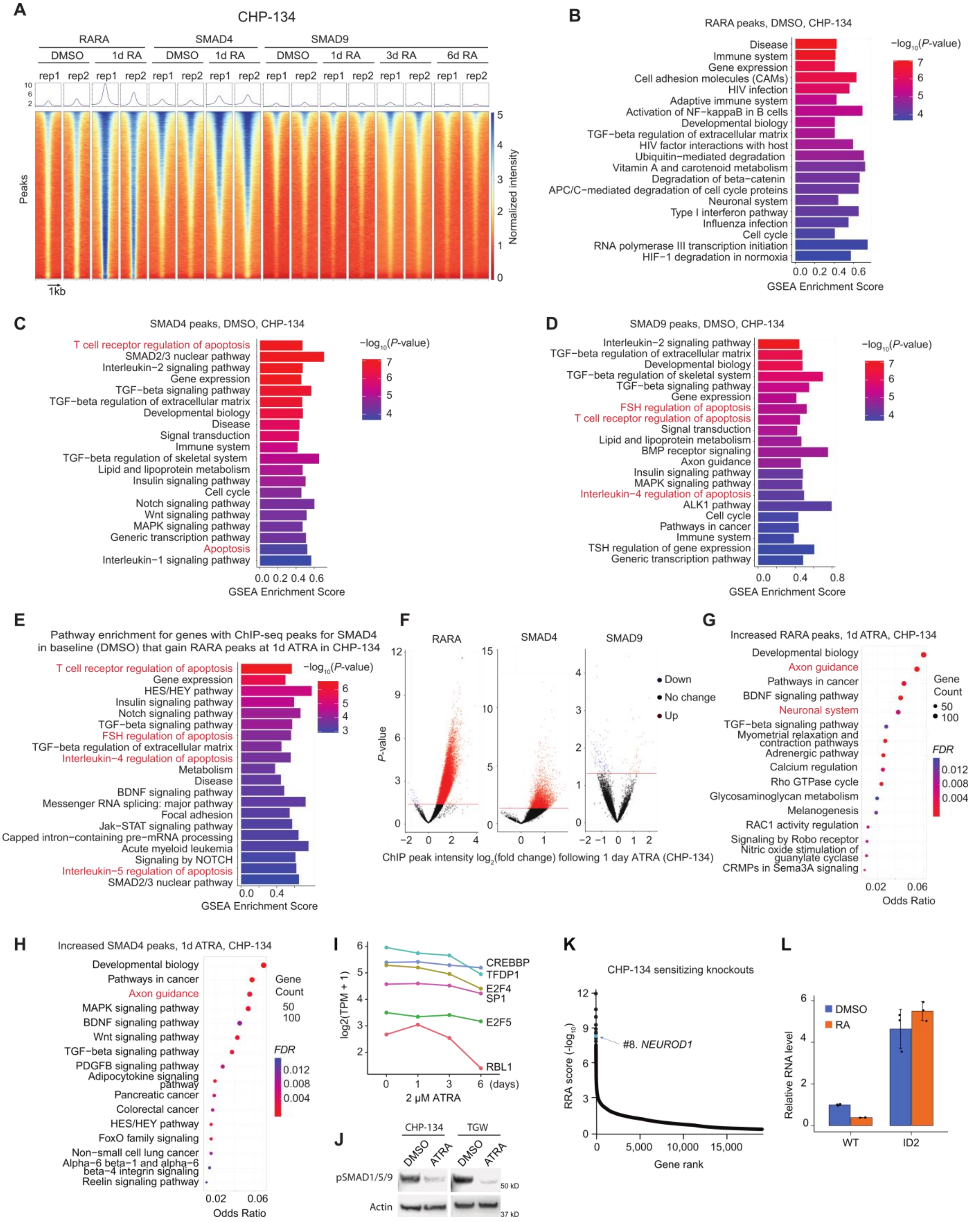
A
CHP-134
RARA
SMAD4
SMAD9
DMSO
1d RA
DMSO
1d RA
DMSO
1d RA
3d RA
6d RA
rep1 rep2 rep1 rep2 rep1 rep2 rep1 rep2 rep1 rep2 rep1 rep2 rep1 rep2 rep1 rep2
Peaks
Normalized intensity
1kb
B
RARA peaks, DMSO, CHP-134
Disease
Immune system
Gene expression
Cell adhesion molecules (CAMs)
HIV infection
Adaptive immune system
Activation of NF−kappaB in B cells
Developmental biology
TGF−beta regulation of extracellular matrix
HIV factor interactions with host
Ubiquitin−mediated degradation
Vitamin A and carotenoid metabolism
Degradation of beta−catenin
APC/C−mediated degradation of cell cycle proteins
Neuronal system
Type I interferon pathway
Influenza infection
Cell cycle
RNA polymerase III transcription initiation
HIF−1 degradation in normoxia
GSEA Enrichment Score
−log10(P-value)
C
SMAD4 peaks, DMSO, CHP-134
T cell receptor regulation of apoptosis
SMAD2/3 nuclear pathway
Interleukin−2 signaling pathway
Gene expression
TGF−beta signaling pathway
TGF−beta regulation of extracellular matrix
Developmental biology
Disease
Signal transduction
Immune system
TGF−beta regulation of skeletal system
Lipid and lipoprotein metabolism
Insulin signaling pathway
Cell cycle
Notch signaling pathway
Wnt signaling pathway
MAPK signaling pathway
Generic transcription pathway
Apoptosis
Interleukin−1 signaling pathway
GSEA Enrichment Score
−log10(P-value)
D
SMAD9 peaks, DMSO, CHP-134
Interleukin−2 signaling pathway
TGF−beta regulation of extracellular matrix
Developmental biology
TGF−beta regulation of skeletal system
TGF−beta signaling pathway
Gene expression
FSH regulation of apoptosis
T cell receptor regulation of apoptosis
Signal transduction
Lipid and lipoprotein metabolism
BMP receptor signaling
Axon guidance
Insulin signaling pathway
MAPK signaling pathway
Interleukin−4 regulation of apoptosis
ALK1 pathway
Cell cycle
Pathways in cancer
Immune system
TSH regulation of gene expression
Generic transcription pathway
GSEA Enrichment Score
−log10(P-value)
E
Pathway enrichment for genes with ChIP-seq peaks for SMAD4 in baseline (DMSO) that gain RARA peaks at 1d ATRA in CHP-134
T cell receptor regulation of apoptosis
Gene expression
HES/HEY pathway
Insulin signaling pathway
Notch signaling pathway
TGF−beta signaling pathway
FSH regulation of apoptosis
TGF−beta regulation of extracellular matrix
Interleukin−4 regulation of apoptosis
Metabolism
Disease
BDNF signaling pathway
Messenger RNA splicing: major pathway
Focal adhesion
Jak−STAT signaling pathway
Capped intron−containing pre−mRNA processing
Acute myeloid leukemia
Signaling by NOTCH
Interleukin−5 regulation of apoptosis
SMAD2/3 nuclear pathway
GSEA Enrichment Score
−log10(P-value)
F
RARA
SMAD4
SMAD9
P-value
Down
No change
Up
ChIP peak intensity log2(fold change) following 1 day ATRA (CHP-134)
G
Increased RARA peaks, 1d ATRA, CHP-134
Developmental biology
Axon guidance
Pathways in cancer
BDNF signaling pathway
Neuronal system
TGF−beta signaling pathway
Myometrial relaxation and contraction pathways
Adrenergic pathway
Calcium regulation
Rho GTPase cycle
Glycosaminoglycan metabolism
Melanogenesis
RAC1 activity regulation
Signaling by Robo receptor
Nitric oxide stimulation of guanylate cyclase
CRMPs in Sema3A signaling
Gene Count
50
100
FDR
Odds Ratio
H
Increased SMAD4 peaks, 1d ATRA, CHP-134
Developmental biology
Pathways in cancer
Axon guidance
MAPK signaling pathway
BDNF signaling pathway
Wnt signaling pathway
TGF−beta signaling pathway
PDGFB signaling pathway
Adipocytokine signaling pathway
Pancreatic cancer
Colorectal cancer
HES/HEY pathway
FoxO family signaling
Non−small cell lung cancer
Alpha−6 beta−1 and alpha−6 beta−4 integrin signaling
Reelin signaling pathway
Gene Count
50
100
FDR
Odds Ratio
I
log2(TPM + 1)
CREBBP
TFDP1
E2F4
SP1
E2F5
RBL1
(days)
2 μM ATRA
J
CHP-134
TGW
DMSO
ATRA
DMSO
ATRA
pSMAD1/5/9
Actin
50 kD
37 kD
K
CHP-134 sensitizing knockouts
#8. NEUROD1
RRA score (-log10)
Gene rank
L
DMSO
RA
Relative RNA level
WT
ID2

Supplementary Figure 5: Additional analyses of ChIP-seq binding profiles in baseline and ATRA treated CHP-134 cells.

A. Like Fig. 5A but SMAD9 ChIP-seq data from 3-day and 6-day treated (2 µM ATRA) samples are included. Genome-wide ChIP-seq binding intensity of RARA, SMAD4, and SMAD9 in CHP-134 cells following treatment with DMSO control or 2 µM ATRA for 1 day, 3 days, or 6 days are shown. Each row represents a genomic region, sorted by average binding intensity across all transcription factors. The color scale represents binding intensity, scale between 0 and 5 (see Methods). The binding profile (rows) of each sample was centered on a binding peak and shows 1kb upstream and downstream sequences from the peak.
B. Bar plot of GSEA enrichment scores (x axis) for BioPlanet pathways in DMSO treated CHP-134 cells, ranking genes based on the intensity of their RARA ChIP-seq peaks. The pathways are ordered by their *P*-value (color scale).
C. Like (B), but for SMAD4.
D. Like (B), but for SMAD9.
E. Bar plot (left panel) shows top GSEA enriched BioPlanet pathways for SMAD4 bound genes that gain RARA peaks following 2 µM ATRA treatment for 1 day (compared to DMSO) in CHP-134 cells.
F. Volcano plot of the changes in peak intensity of RARA, SMAD4, and SMAD9 in CHP-134 cells following 2 µM ATRA exposure for 1 day (compared to DMSO). Peaks with a $\log_2$ fold change (x axis) of >1 or < -1 and an adjusted *P*-value < 0.05 are highlighted as significant (colors).
G. Dot plot of the top enriched pathways for genes whose RARA peak intensity increased with 2 µM ATRA treatment for 1 day (compared to DMSO). *P*-values and odds ratios (x axis) were calculated by hypergeometric test.
H. Like (G), except for genes whose SMAD4 peak intensity increased with RA treatment.
I. Line plot of the expression changes of TGF-β signaling target genes regulated by SMAD2/3. The log2-transformed TPM values of CHP-134 cells, following treatment with DMSO control or 2 µM ATRA for 1 day, 3 days, or 6 days, are shown.
J. Western blots showing expression of pSMAD1/5/9 in CHP-134 and TGW cells treated with 2 µM ATRA for 3 days. Representative results of 3 independent replicates are shown.
K. Waterfall plot showing top hits involved in BMP signaling, ranked by negative RRA score (a higher value on y-axis indicates the gene knockout is more likely to increase CHP-134 sensitivity to ATRA). *NEUROD1* is highlighted.
L. *ID2* expression in wild-type (WT) and *ID2*-overexpressed (*ID2*) CHP-134 cells following DMSO or 2 µM RA for 6 days. Data represents the mean ± SD of 3 independent replicates (n = 3). *P*-values are included in the Source Data file.

Source data are provided as a Source Data file.

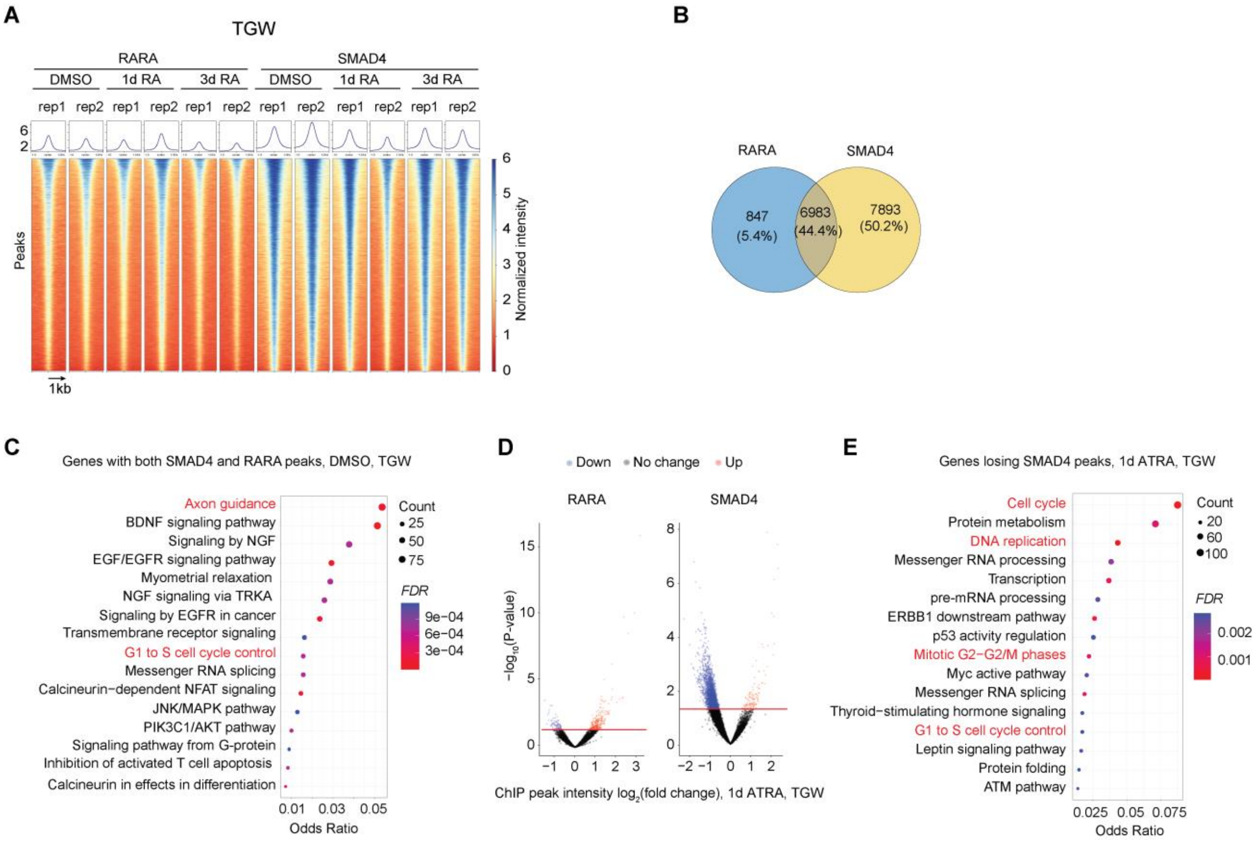

Supplementary Figure 6: Additional analyses of ChIP-seq binding profiles in baseline and ATRA treated TGW cells.

A. Heatmap showing RARA and SMAD4 binding ChIP-seq profiles in TGW cells. The cells were treated with DMSO, 2 µM ATRA for 1 day, or 0.5 µM ATRA for 3 days. Two independent replicates are shown.
B. Venn diagram showing the number and proportion of overlapping binding peaks between RARA and SMAD4 in TGW.
C. Dot plot showing the most enriched pathways for genes bound by both SMAD4 and RARA in DMSO treated TGW cells. *P*-values and odds ratios (x axis) were calculated by hypergeometric test.
D. Volcano plot showing intensity change of RARA and SMAD4 binding peaks in TGW cells in response to 2 µM ATRA exposure for 1 day (compared to DMSO). Peaks with a $\log_2$ fold change > 1 or < -1 and an *FDR* <0.05 were considered significant (colors).
E. Dot plot showing the most enriched pathways for genes whose SMAD4 peak intensity decreased significantly with 2 µM ATRA treatment for 1 day. *P*-values and odds ratios (x axis) were calculated by hypergeometric test.

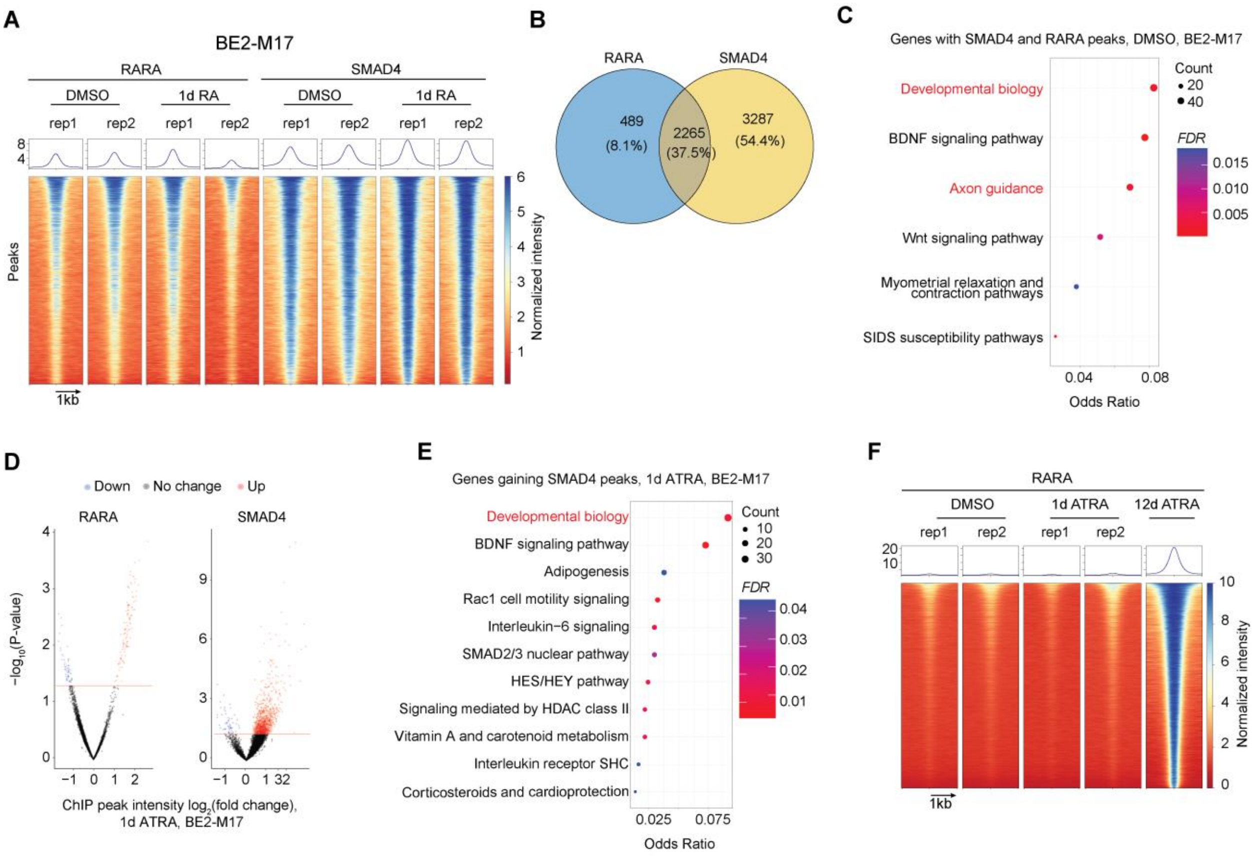

Supplementary Figure 7: Additional analyses of ChIP-seq binding profiles in baseline and ATRA treated BE(2)-M17 cells.

A. Heatmap showing RARA and SMAD4 binding profiles for all data collected in BE(2)-M17 cells.
B. Venn diagram showing the number and proportion of overlapping binding peaks between RARA and SMAD4 in BE(2)-M17.
C. Dot plot showing the most enriched pathways for genes bound by both SMAD4 and RARA in baseline DMSO treated BE(2)-M17 cells. *P*-values and odds ratios (x axis) calculated by hypergeometric test.
D. Volcano plot showing changes in peak intensity of RARA and SMAD4 in BE(2)-M17 cells in response to 2 µM ATRA exposure for 1 day. Peaks with a $\log_2$ fold change > 1 or < -1 and an FDR <0.05 were considered significant (colors).
E. Dot plot showing the most enriched pathways for genes whose SMAD4 peak intensity increased significantly with 2 µM ATRA treatment for 1 day in BE(2)-M17 cells. *P*-values and odds ratios (x axis) calculated by hypergeometric test.
F. Heatmap displaying the genome-wide ChIP-seq binding intensity of RARA in BE(2)-M17 following treatment with DMSO control or 2 µM ATRA for 1 d, in comparison to the RARA binding profile in BE2C cell line treated with 5 µM ATRA for 12 days, where these cells eventually gain RARA peaks.

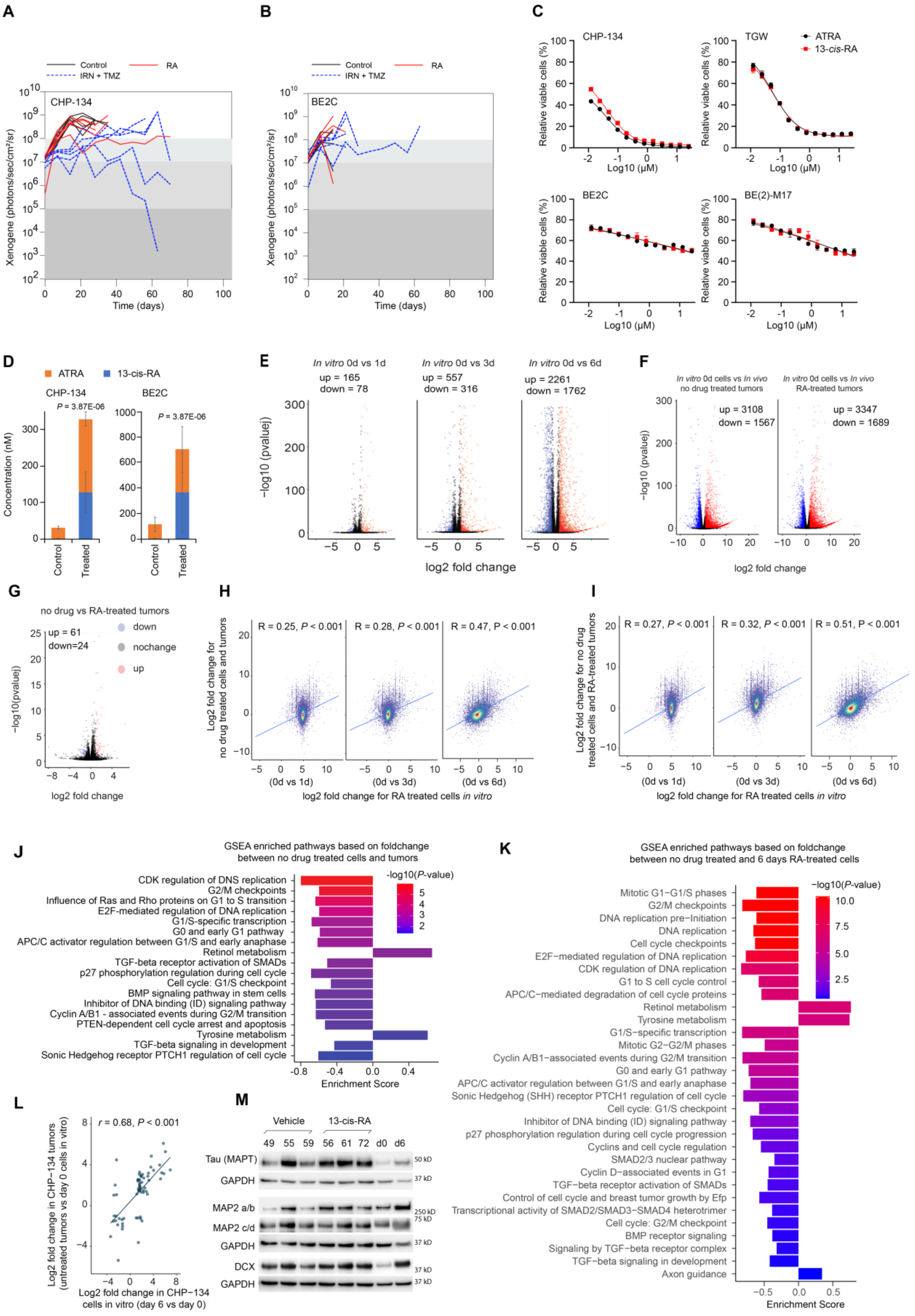
A
Control
RA
IRN + TMZ
CHP-134
Xenogene (photons/sec/cm²/sr)
Time (days)
B
Control
RA
IRN + TMZ
BE2C
Xenogene (photons/sec/cm²/sr)
Time (days)
C
CHP-134
TGW
ATRA
13-cis-RA
BE2C
BE(2)-M17
Relative viable cells (%)
Log10 (μM)
D
ATRA
13-cis-RA
CHP-134
BE2C
P = 3.87E-06
P = 3.87E-06
Concentration (nM)
Control
Treated
E
In vitro 0d vs 1d
up = 165
down = 78
In vitro 0d vs 3d
up = 557
down = 316
In vitro 0d vs 6d
up = 2261
down = 1762
−log10 (pvalue)
log2 fold change
F
In vitro 0d cells vs In vivo no drug treated tumors
In vitro 0d cells vs In vivo RA-treated tumors
up = 3108
down = 1567
up = 3347
down = 1689
−log10 (pvalue)
log2 fold change
G
no drug vs RA-treated tumors
up = 61
down=24
down
nochange
up
−log10(pvalue)
log2 fold change
H
R = 0.25, P < 0.001
R = 0.28, P < 0.001
R = 0.47, P < 0.001
Log2 fold change for no drug treated cells and tumors
(0d vs 1d)
(0d vs 3d)
(0d vs 6d)
log2 fold change for RA treated cells in vitro
I
R = 0.27, P < 0.001
R = 0.32, P < 0.001
R = 0.51, P < 0.001
Log2 fold change for no drug treated cells and RA-treated tumors
(0d vs 1d)
(0d vs 3d)
(0d vs 6d)
log2 fold change for RA treated cells in vitro
J
GSEA enriched pathways based on foldchange between no drug treated cells and tumors
CDK regulation of DNS replication
G2/M checkpoints
Influence of Ras and Rho proteins on G1 to S transition
E2F-mediated regulation of DNA replication
G1/S-specific transcription
G0 and early G1 pathway
APC/C activator regulation between G1/S and early anaphase
Retinol metabolism
TGF-beta receptor activation of SMADs
p27 phosphorylation regulation during cell cycle
Cell cycle: G1/S checkpoint
BMP signaling pathway in stem cells
Inhibitor of DNA binding (ID) signaling pathway
Cyclin A/B1 - associated events during G2/M transition
PTEN-dependent cell cycle arrest and apoptosis
Tyrosine metabolism
TGF-beta signaling in development
Sonic Hedgehog receptor PTCH1 regulation of cell cycle
-log10(P-value)
Enrichment Score
K
GSEA enriched pathways based on foldchange between no drug treated and 6 days RA-treated cells
Mitotic G1−G1/S phases
G2/M checkpoints
DNA replication pre−Initiation
DNA replication
Cell cycle checkpoints
E2F−mediated regulation of DNA replication
CDK regulation of DNA replication
G1 to S cell cycle control
APC/C−mediated degradation of cell cycle proteins
Retinol metabolism
Tyrosine metabolism
G1/S−specific transcription
Mitotic G2−G2/M phases
Cyclin A/B1−associated events during G2/M transition
G0 and early G1 pathway
APC/C activator regulation between G1/S and early anaphase
Sonic Hedgehog (SHH) receptor PTCH1 regulation of cell cycle
Cell cycle: G1/S checkpoint
Inhibitor of DNA binding (ID) signaling pathway
p27 phosphorylation regulation during cell cycle progression
Cyclins and cell cycle regulation
SMAD2/3 nuclear pathway
Cyclin D−associated events in G1
TGF−beta receptor activation of SMADs
Control of cell cycle and breast tumor growth by Efp
Transcriptional activity of SMAD2/SMAD3−SMAD4 heterotrimer
Cell cycle: G2/M checkpoint
BMP receptor signaling
Signaling by TGF−beta receptor complex
TGF−beta signaling in development
Axon guidance
-log10(P-value)
Enrichment Score
L
r = 0.68, P < 0.001
Log2 fold change in CHP−134 tumors (untreated tumors vs day 0 cells in vitro)
Log2 fold change in CHP−134 cells in vitro (day 6 vs day 0)
M
Vehicle
13-cis-RA
49 55 59 56 61 72 d0 d6
Tau (MAPT)
GAPDH
MAP2 a/b
MAP2 c/d
GAPDH
DCX
GAPDH
50 kD
37 kD
250 kD
75 kD
37 kD
37 kD
37 kD

Supplementary Figure 8

A. Mice were implanted with CHP-134 cells. Figure shows the photon emissions of the resulting tumors (as measured by the Xenogen in vivo imaging system). Greater photon emissions indicate greater tumor volume. Curves are colored by the treatment group.
B. Same as in (A), but the tumors were generated by implanting mice with BE2C cells.
C. Viability of cell lines (CHP-134, TGW, BE2C, BE(2)-M17) treated for 6 days with *all-trans*-retinoic acid (ATRA) or 13-cis-retinoic acid (13-*cis*-RA). Data represents the mean ± SD of 3 independent experiments (n = 3). *P*-values are included in the Source Data file.
D. Concentrations of ATRA and 13-*cis*-RA in vehicle (Control) and 13-*cis*-RA-treated (Treated) tumor tissues. Tumors were collected within two weeks of the last treatment. The number of tumors in group are as follows: four in CHP-134 Control; three in CHP-134 Treated; five in BE2C Control; and four in BE2C Treated. P-values were calculated using two-tailed t tests. *P*-values are included in the Source Data file.
E. Volcano plot of the differentially expressed genes in CHP-134 cells treated with RA for 1, 3, and 6 days versus control CHP-134 cells (0 days). Cells were cultured *in vitro.* Genes were considered differentially expressed if they exhibited a log2 fold change greater than 1 or less than -1 as well as an adjusted p-value of less than 0.05.
F. Left: volcano plot of the differentially expressed genes in untreated CHP-134 tumors vs untreated CHP-134 cells cultured *in vitro*. Right: volcano plot of the differentially expressed genes in CHP-134 tumors treated with RA vs untreated CHP-134 cells cultured in *vitro.*
G. Volcano plot of the differentially expressed genes in RA-treated vs untreated (control) CHP-134 tumors.
H. Scatter plots comparing the gene expression fold change between untreated CHP-134 cells cultured *in vitro* and untreated CHP-134 tumors versus the gene expression fold change between untreated CHP-134 cells cultured *in vitro* and CHP-134 cells treated with RA for 1 day (left panel), 3 days (middle panel) and 6 days (right panel) *in vitro*. Pearson correlation coefficients and p-values are displayed on the plots.
I. Similar to (I), but the values on the y-axis represent the fold change between untreated CHP-134 cells cultured *in vitro* and RA-treated CHP-134 tumors.
J. Bar plot of the enriched pathways identified through GSEA analysis. In this analysis, genes were ranked by the fold change in their expression in untreated CHP-134 cells cultured *in vitro* versus untreated CHP-134 tumors.
K. Bar plot of the enriched pathways identified through GSEA analysis. In this analysis, genes were ranked by the fold change in their expression in untreated CHP-134 cells cultured *in vitro* versus CHP-134 cells treated with RA for 6 days *in vitro*.
L. For differentially expressed genes enriched in axon guidance pathway, as shown in Fig. 6E, their expression fold change between untreated CHP-134 cells cultured *in vitro* and untreated CHP-134 tumors *in vivo* are plotted against the expression fold change between untreated CHP-134 cells cultured *in vitro* and CHP-134 cells treated with RA for 6 days. Pearson correlation coefficients and *P*-values are displayed on the plots.
M. Western blots showing the expression of neuron markers MAPT, MAP2, and DCX in CHP-134 xenograft tumor tissues and the cell line cultured in vitro (treated with DMSO (d0) or 2 µM RA for 6 days (d6)). GAPDH was used loading control. All the samples were derived from the same experiment and the same samples were ran in different gels

for MAPT, MAP2, and DCX. The representative results of 3 independent replicates are shown.

Source data are provided as a Source Data file.

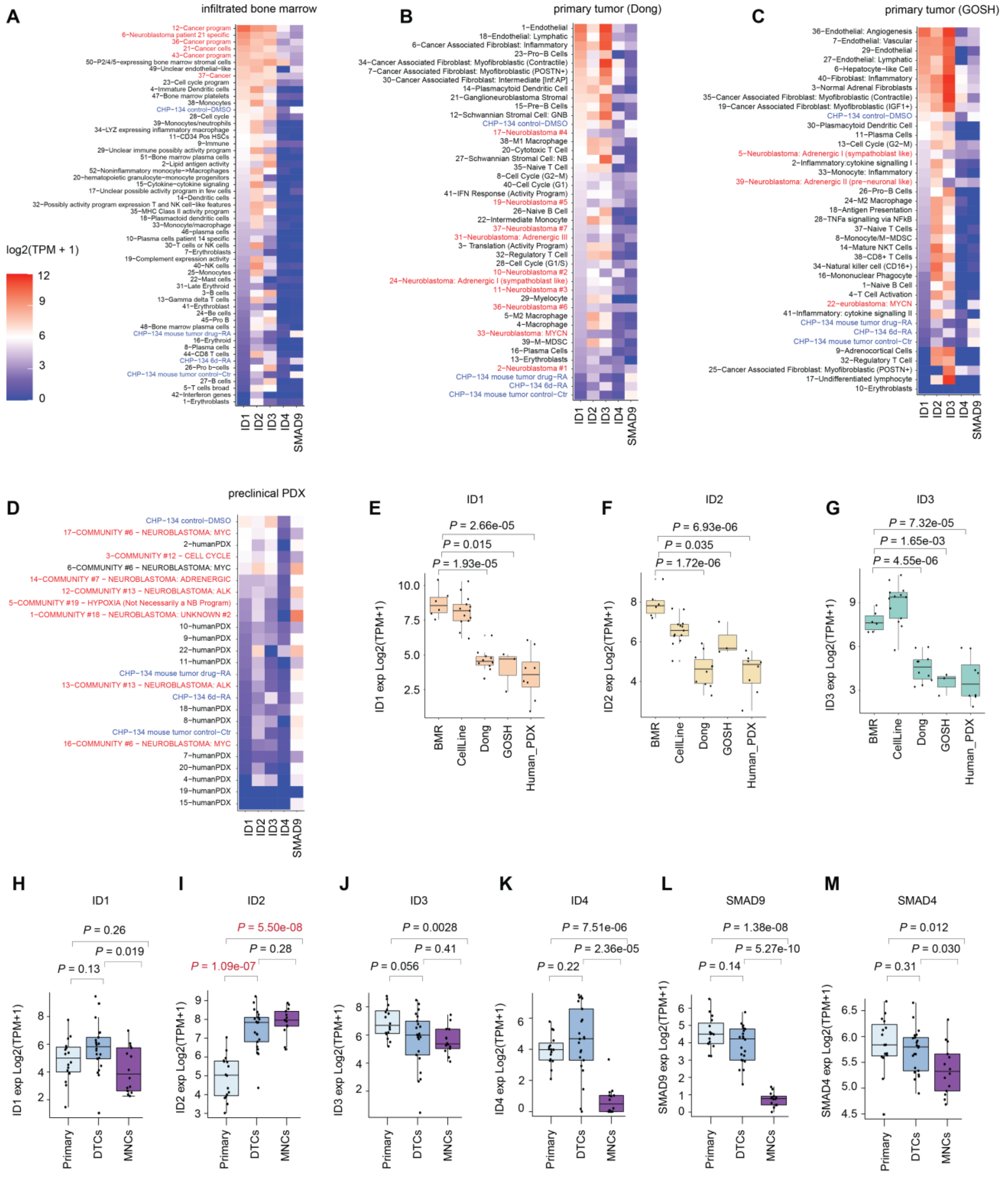

Supplementary Figure 9

A. Heatmap showing the expression levels of *ID* genes and *SMAD9* in cells expression 52 gene expression programs (cell clusters categorized by gene expression profiles) identified from single-cell RNA-sequencing data of neuroblastoma-infiltrated bone

marrow samples (data from Fetahu *et al*, PMID 37365178). Programs highlighted in red represent those expressed in neuroblastoma cancer cells, while those in black indicate tumor-associated normal cells. Blue represents CHP-134 cells and *in vivo* tumors, which have been integrated into the plot to serve as a reference for expression comparison with the bone marrow cancer cells.

B. Similar to (A) but for single-cell RNA-seq in primary neuroblastoma tumors (data from Dong et al, PMID 32946775).
C. Similar to (A) but for single-cell RNA-seq in another primary neuroblastoma dataset (data from Kildisuite *et al*, PMID 33547074).
D. Similar to (A) but for single-cell RNA-seq of a preclinical PDX dataset (data from Chapple *et al*.).
E. Boxplots showing the expression of *ID1* in cells expressing the cancer programs (programs highlight by red color in A-D) identified in single-cell RNA-seq from bone marrow (BMR), human primary neuroblastoma tumors (Dong and GOSH), PDXs model (Human PDX) and 13 neuroblastoma cell lines (Cell line).
F. Similar to (E) but for the expression of *ID2*.
G. Similar to (E) but for the expression of *ID3*.
H. Boxplots (H-M) showing $\log_2(TPM + 1)$ normalized bulk RNA-seq expression (y axis) of key BMP pathway genes *ID1-4* (H-K), *SMAD9* (L) and *SMAD4* (M) in primary human neuroblastoma tumors and bone marrow metastatic sites from the same patients. Boxes represent bulk primary tumors (‘Primary’; light blue), disseminated tumors cells (‘DTCs’; dark blue) in the bone marrow enriched using an anti-GD2 antibody, and the remaining mononuclear cells (‘MNCs’; purple) following depletion of GD2 expressing cells. Data were obtained Rifatbegovic *et al*. (PMID 28921546).

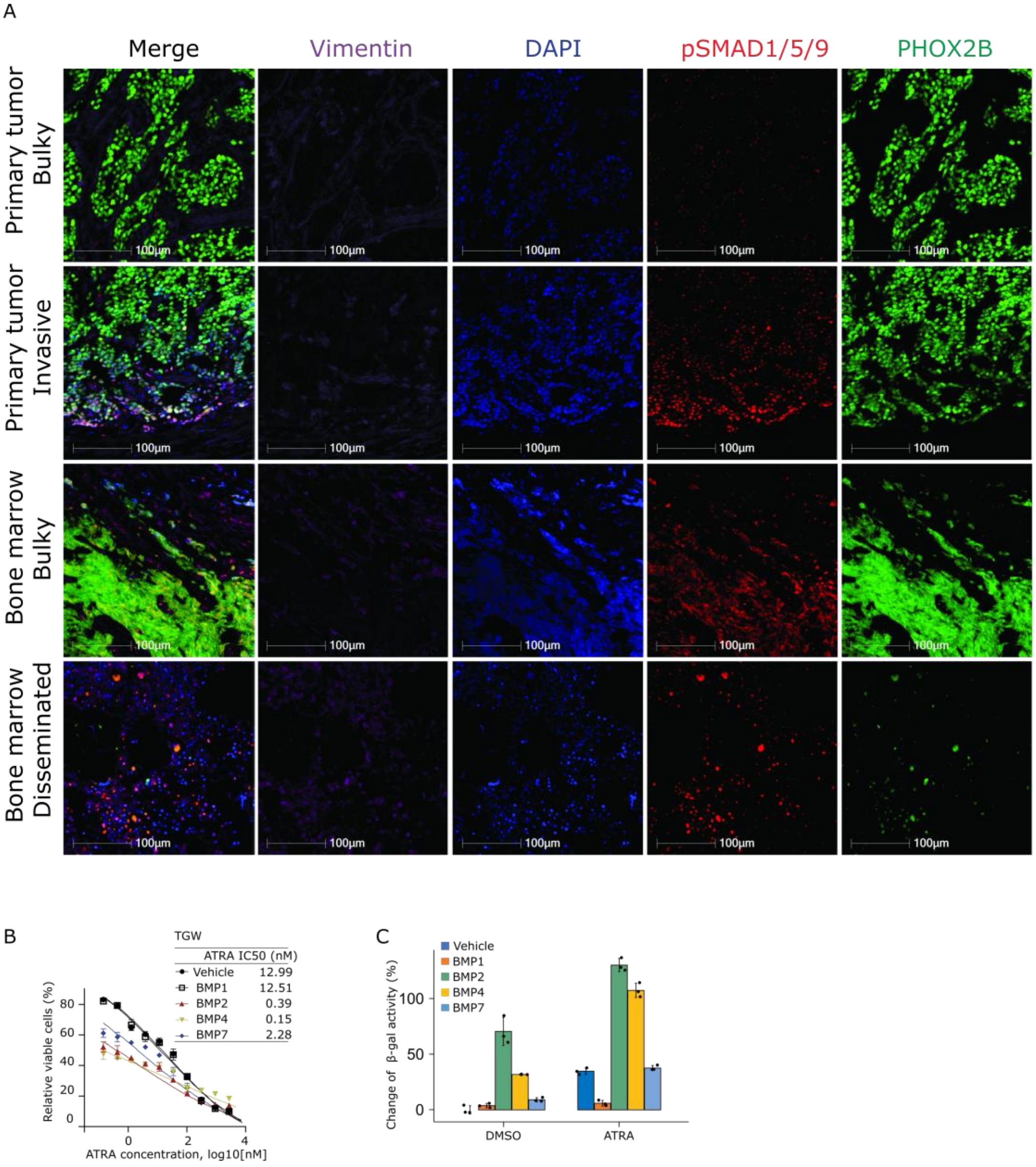

Supplementary Figure 10

A. Related to Fig. 6H. Immunofluorescent images from Patient #3 showing Vimentin, DAPI, pSMAD1/5/9, and PHOX2B staining. The cells co-stained with pSMAD1/5/9 and PHOX2B appear yellow or orange in the merged images.
B. Viability of TGW cells treated with ATRA for 6 days in the presence of vehicle control or 10 ng/ml BMP recombinant proteins. Cell viability was measured with CellTiter-Glo. ATRA IC50 for TGW (right) in the presence of each BMP was calculated with GraphPad Prism 9. Data represent the mean ± SD of 3 independent replicates (n = 3).
C. Senescence levels quantified by intensity of fluorescence in TGW cells treated with DMSO and 0.03 µM ATRA for 6 days in the presence of vehicle control or 10 ng/ml BMP recombinant proteins. Data represent the mean ± SD of 3 independent replicates (n = 3).

For panel B and C, *P*-values are included in the Source Data file. Source data are provided as a Source Data file.

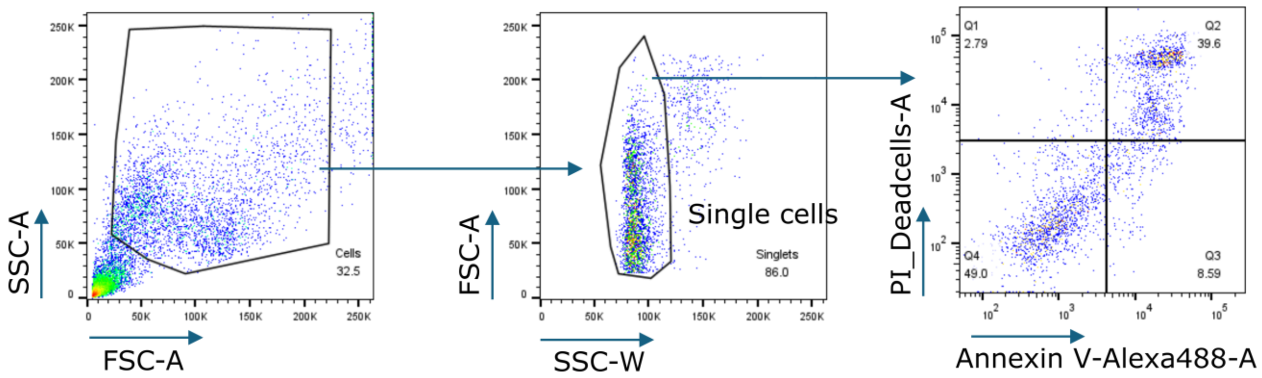

Supplementary Figure 11

Gating strategy for flow cytometry analysis in this study.

Figure 1C

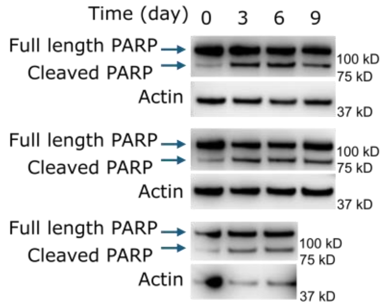

Figure 1G

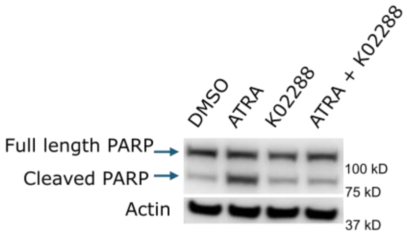

Figure S1K

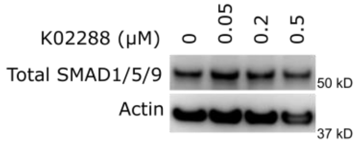

Figure 2D

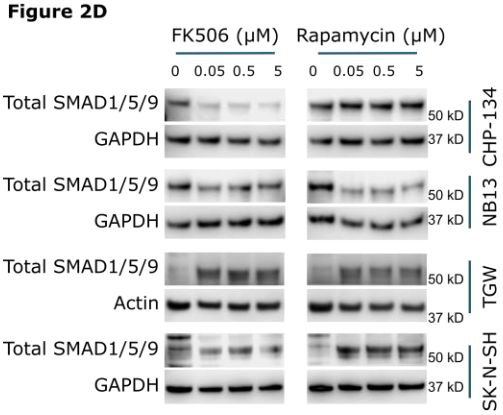

Figure 3C

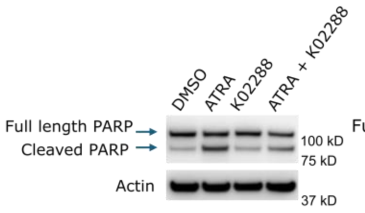

Figure 3G

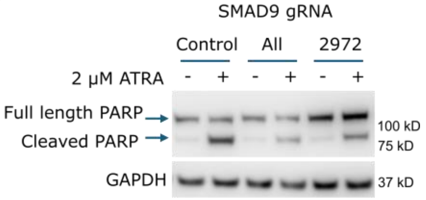

Figure 3H

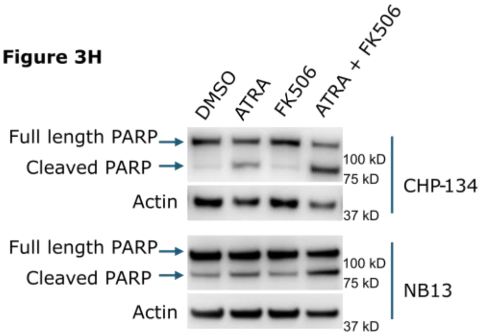

Figure S3A

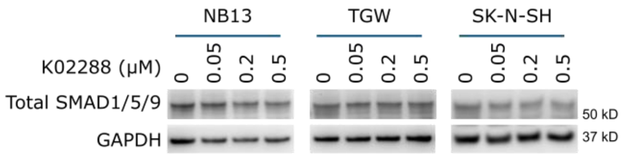

Figure 5I

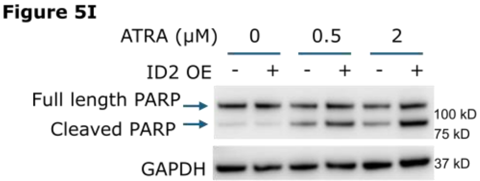

Figure S5J

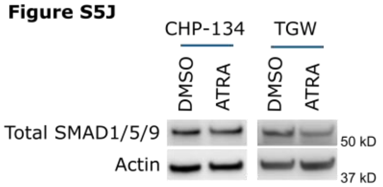

Figure 6D

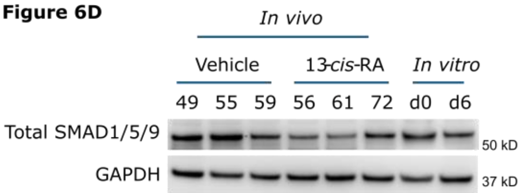

Figure 6J

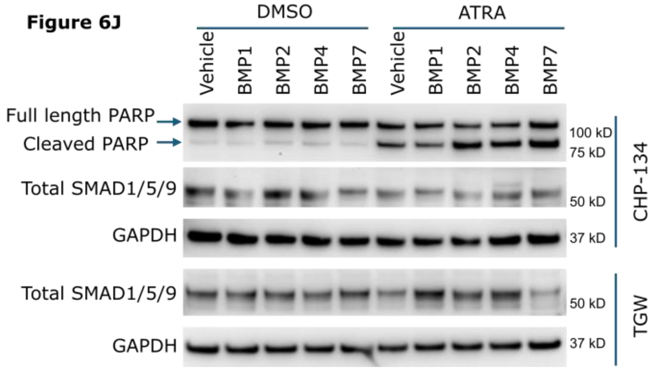

Supplementary Figure 12

Western blots showing full length PARP, cleaved PARP, and total SMAD1/5/9 expression for the indicated figures.

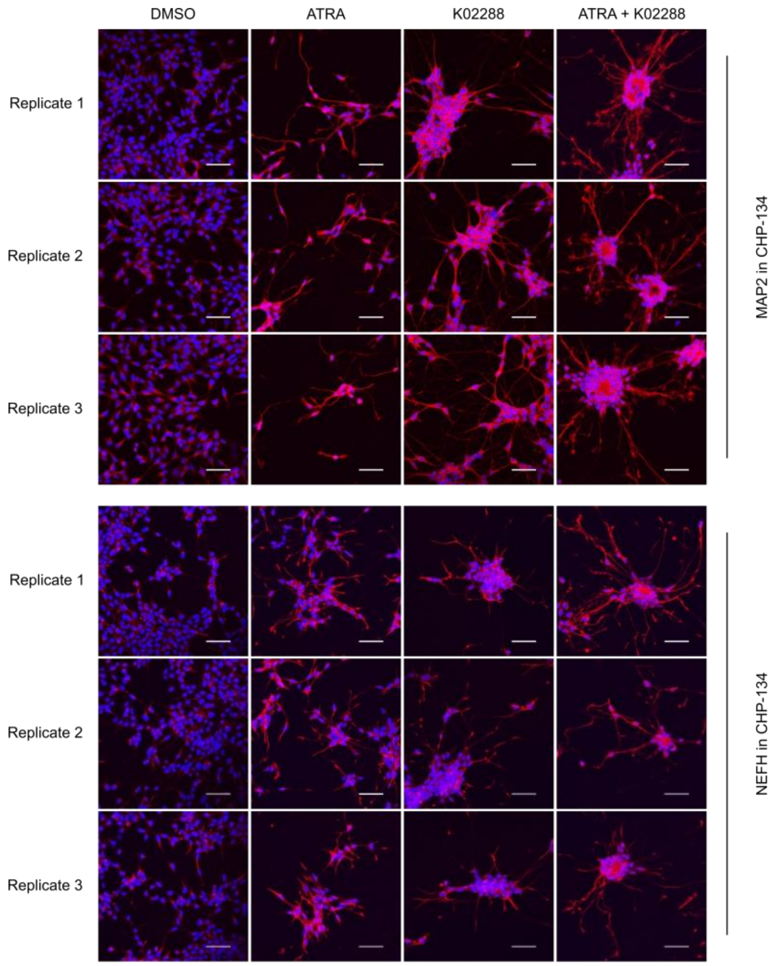
DMSO
ATRA
K02288
ATRA + K02288
Replicate 1
Replicate 2
Replicate 3
MAP2 in CHP-134
Replicate 1
Replicate 2
Replicate 3
NEFH in CHP-134

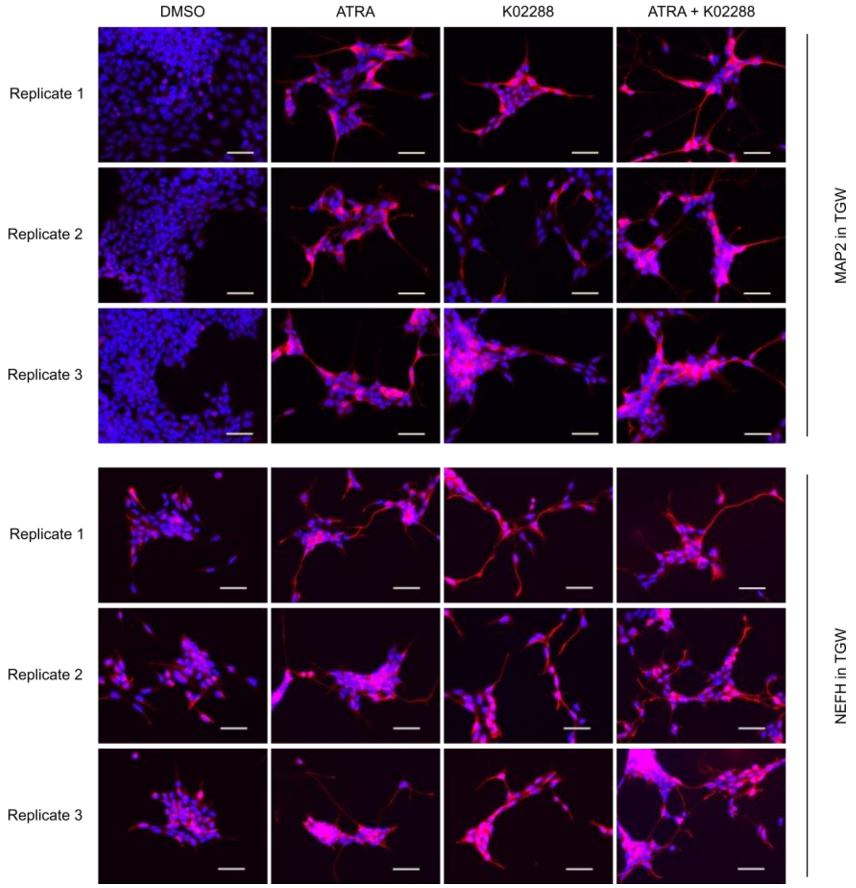
DMSO
ATRA
K02288
ATRA + K02288
Replicate 1
Replicate 2
Replicate 3
MAP2 in TGW
Replicate 1
Replicate 2
Replicate 3
NEFH in TGW

Supplementary Figure 13

Related to Figure 3A and 4A. Immunofluorescent images showing expression of MAP2 and NEFH in CHP-134 and TGW cells. Blue, DAPI; red, MAP2/NEFH.

Supplementary Figure 14

IF images of the patient samples shown in Fig. 6G – H. 25x magnification. Images were taken with a Zeiss Axioscan slide scanner and imaged using HALO v3.6.4134.137 (Indica Labs).

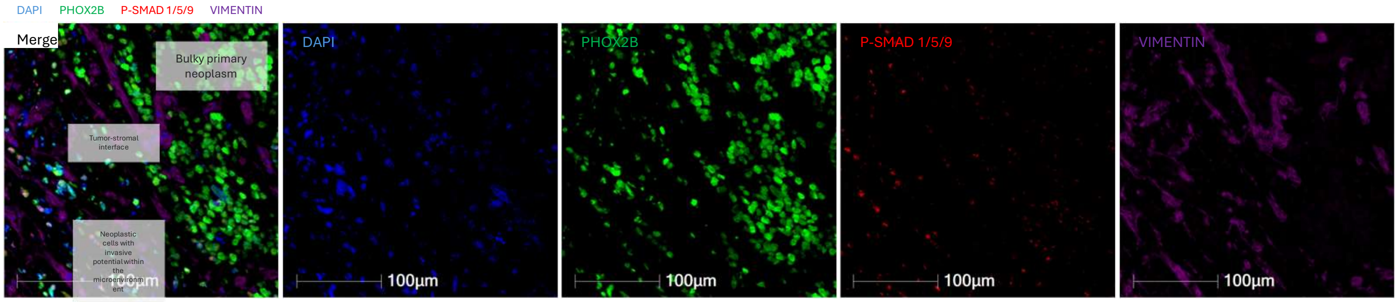

Patient #1 primary tumor: images of primary tumor mass and show that subpopulations of PHOX2B+ve neoplastic cells at the tumor-stromal borders and with presumed increased invasive capabilities visually appear to have increased immunolabeling for p-SMAD1/5/9 by visual estimation.

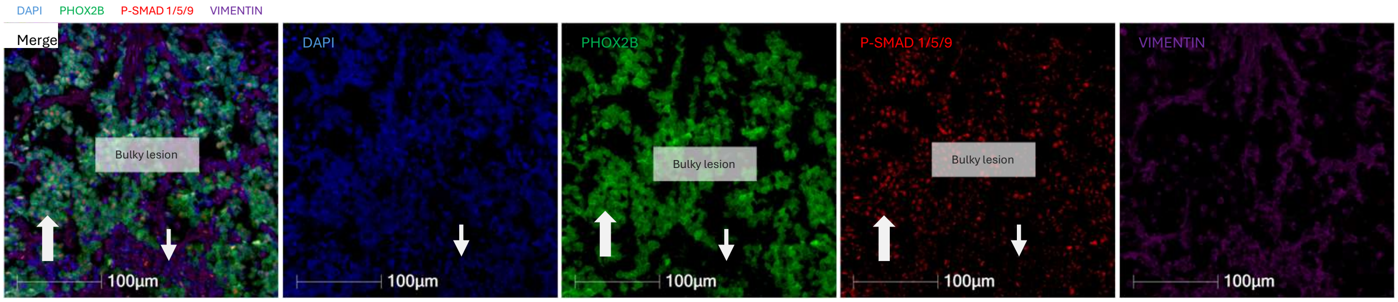

Patient #1 bone marrow: Image of neoplastic cells forming a bulky bone marrow metastatic lesion with interspersed hematopoietic and stroma cells showing immunolabeling for pSMAD1/5/9 in the bulky metastatic lesion (thick arrows). pSMAD1/5/9 is also observed within hematopoietic and stroma cell populations within the bone marrow microenvironment (thin arrows).

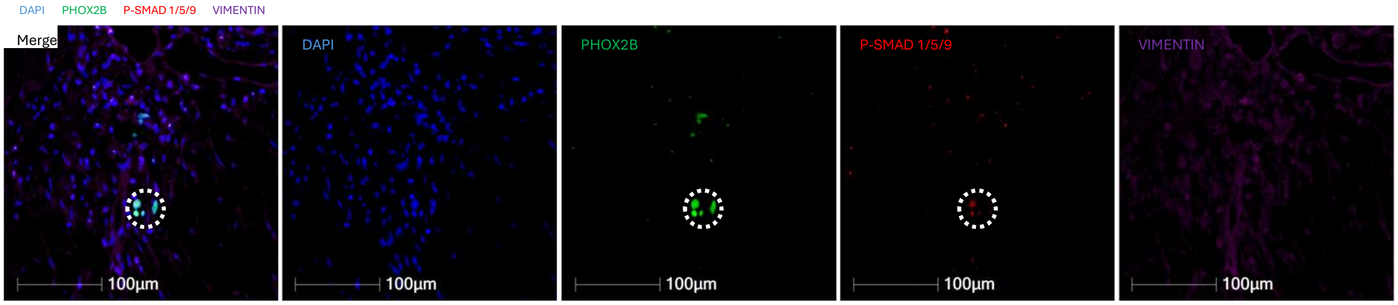

Patient #2 primary tumor: Post-surgical removal of a post-treatment neuroblastoma primary mass showing that subpopulations of residual PHOX2B+ve neoplastic cells retain p-SMAD1/5/9 immunolabeling (dashed circles).

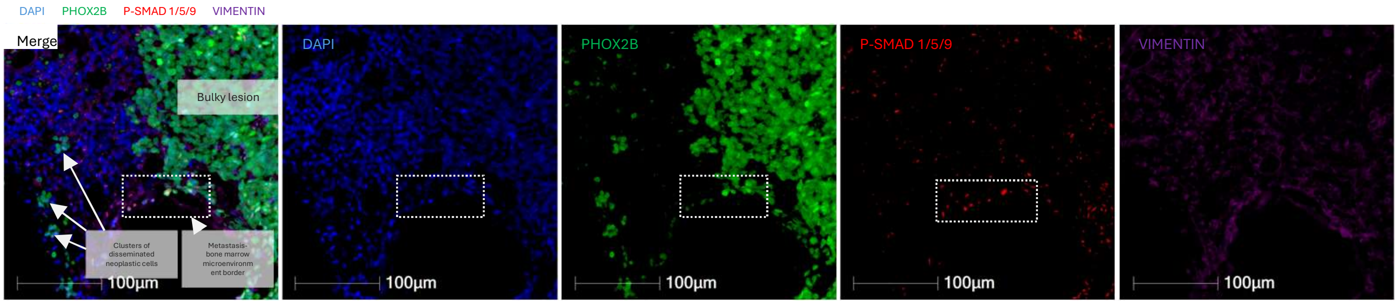

Patient #2 bone marrow: Image of neoplastic cells forming a bulky metastatic lesion with interspersed hematopoietic and stroma cells showing visually increased immunolabeling for pSMAD1/5/9 in invasive neoplastic cells located at the interface with the bone marrow microenvironment (dashed rectangle) when compared with cells forming the bulky metastatic lesion. pSMAD1/5/9 is also observed within hematopoietic and stroma cell populations within the bone marrow microenvironment.

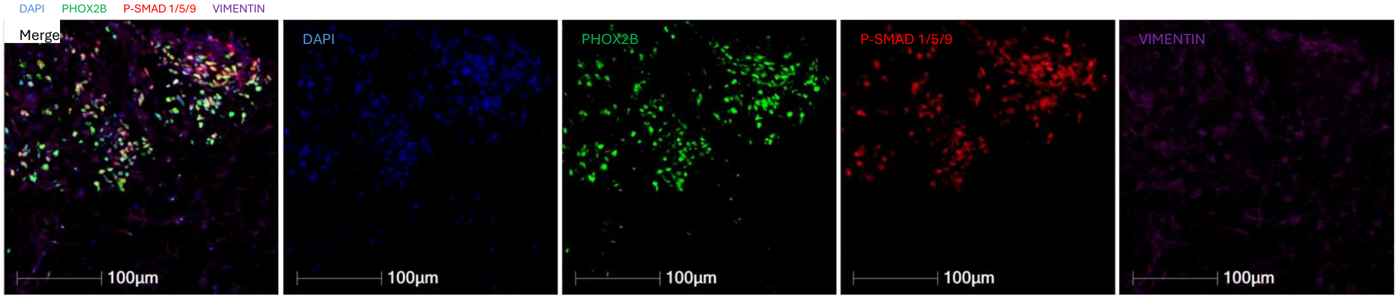

Patient #4 primary tumor: needle biopsy specimen of a neuroblastoma primary mass showing an additional example of increased immunolabeling and staining intensities for pSMAD1/5/9 in subpopulations of PHOX2B+ve neoplastic cells present at tumor-stromal borders. 25x magnification. The bottom panel of images shows foci of lymphovascular invasion.

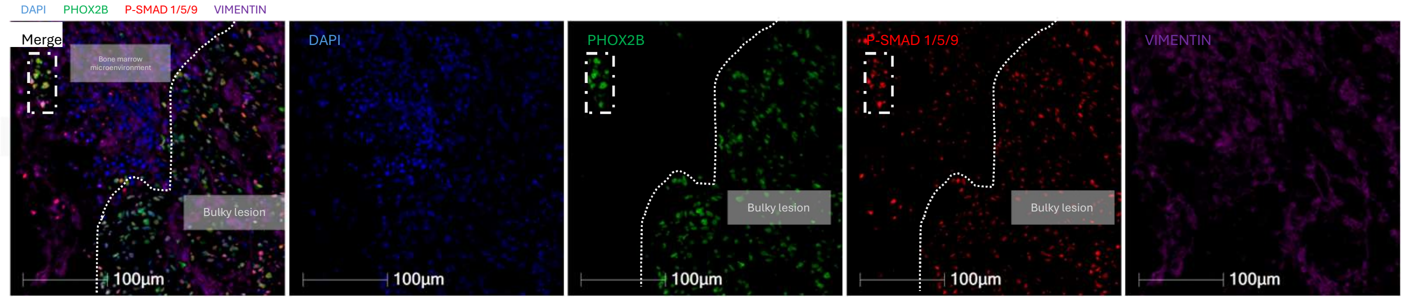

Patient #4 bone marrow: Image from neuroblastoma bone metastases highlighting immunolabeling for pSMAD1/5/9 in PHOX2B+ve neoplastic cells with metastatic capability. Neoplastic cells that form bulky metastatic lesions (freeform shape) have immunolabeling that appears somewhat reduced in staining intensity when compared with an adjacent smaller sized neoplastic embolus (dashed rectangle). Immunolabeling is observed in some cell populations in the bone marrow microenvironment.

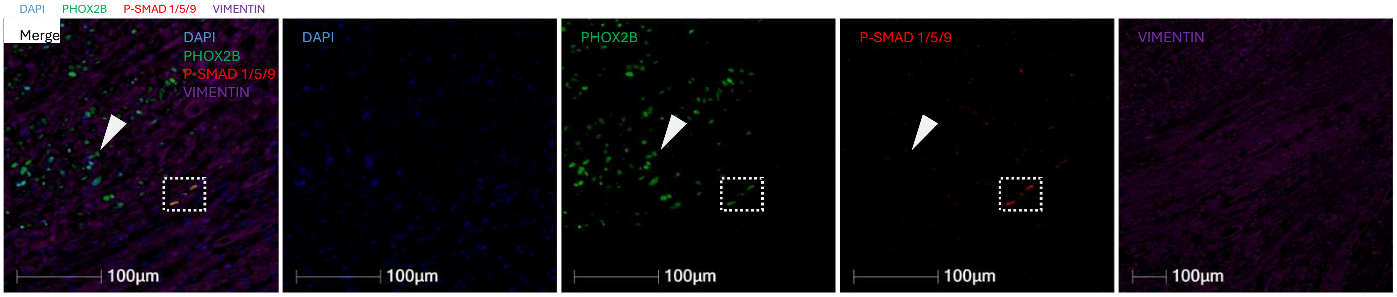

Patient #5 primary tumor: Neuroblastoma primary mass showing that subpopulations of PHOX2B+ve neoplastic cells at the tumor-stromal borders and with presumed increased invasive capabilities visually appear to have increased immunolabeling for p-SMAD1/5/9 (dashed rectangles).

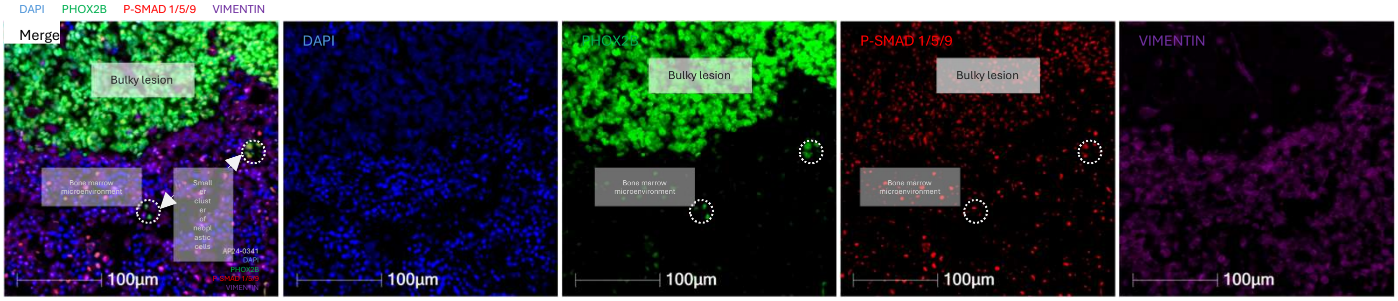

Patient #5 bone marrow: Image from neuroblastoma bone metastases highlighting immunolabeling for pSMAD1/5/9 in PHOX2B+ve neoplastic cells with metastatic capability. Neoplastic cells that form bulky metastatic lesions (freeform shape) have immunolabeling that appears somewhat reduced in staining intensity when compared with an adjacent smaller sized neoplastic cells that are individualized or clustered (dashed circles). Immunolabeling is also observed in some cell populations in the bone marrow microenvironment.

DAPI PHOX2B P-SMAD 1/5/9 VIMENTIN

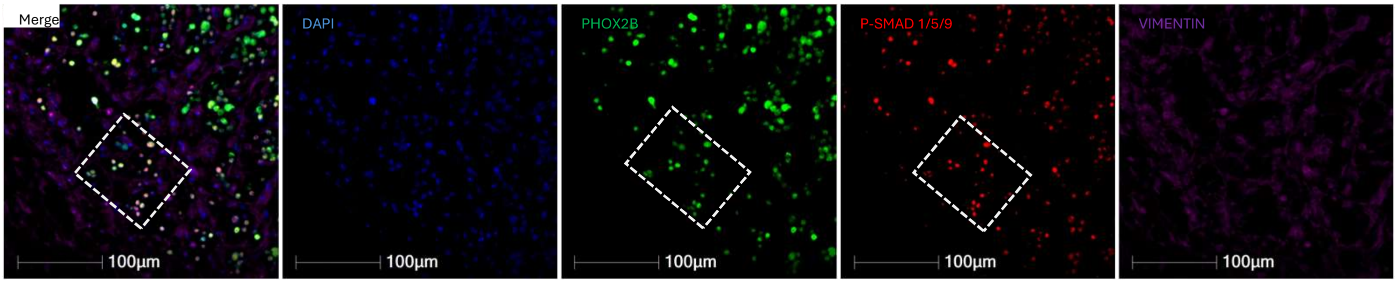

Patient #6 primary tumor: top panel of images are taken from a neuroblastoma primary mass and show that subpopulations of PHOX2B+ve neoplastic cells at the tumor-stromal borders and with presumed increased invasive capabilities visually appear to have increased immunolabeling for p-SMAD1/5/9 (turned and dashed rectangles).

DAPI PHOX2B P-SMAD 1/5/9 VIMENTIN

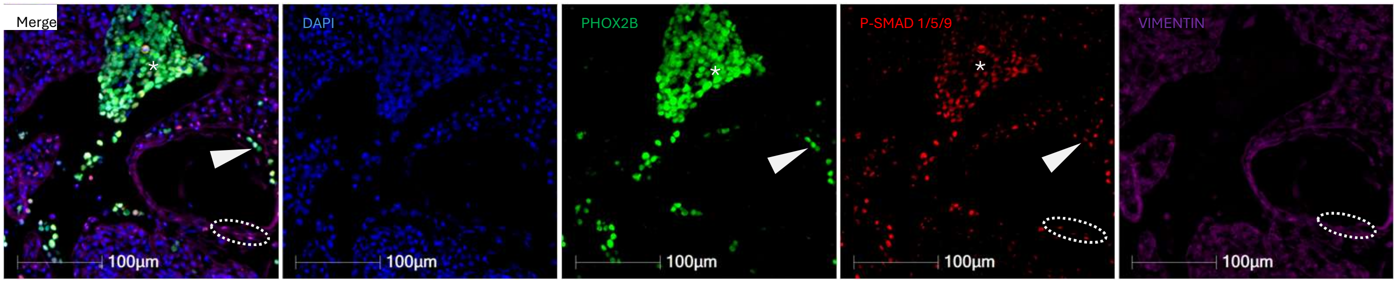

Patient #6 bone marrow: images taken from neuroblastoma bone metastases highlighting that there is increased immunolabeling and staining intensities for pSMAD1/5/9 in PHOX2B+ve neoplastic cells with metastatic capability. Variably sized neoplastic emboli and individualized cells are present within sinusoids (*) and in and around vessels (arrowheads). Dashed oval shows endothelium, which expresses pSMAD 1/5/9, and serves as an internal positive tissue control. 25x magnification. Images were taken with a Zeiss Axioscan slide scanner and imaged using HALO v3.6.4134.137 (Indica Labs).
