# Supplementary material for: Bone morphogenetic protein (BMP) signaling determines neuroblastoma cell fate and sensitivity to retinoic acid
Source: Nat Commun. 2025 Feb 28;16:2036. doi: 10.1038/s41467-025-57185-y (PMC11871043; doi:10.1038/s41467-025-57185-y)
Supplement: Supplementary file 2 — Description of Additional Supplementary Information [file 41467_2025_57185_MOESM2_ESM.docx]

Supplementary Data 1. ATRA IC50, SMAD1/5/9 expression, and drug synergy score in cell lines. SMAD1/5/9 expression data are from GDSC and Depmap. The genetic background of these cell lines is also provided.

Supplementary Data 2. Genome-wide CRISPR knockout screen in CHP-134 cell line. Data from the cells treated for 3 days and 6 days are provided.

Supplementary Data 3. ChIP-seq and RNA-seq data in this study. A summary of sample and data collection is included.

Supplementary Data 4. GSEA analysis results from ChIP-seq data.

Supplementary Data 5. Related to Figure 5e – f. The overrepresentation of RARA and SMAD4 binding genes within the differentially expressed genes.

Supplementary Data 6. Related to Figure 5h. Motif enrichment at RARA binding regions.

Supplementary Data 7. Genomic and clinical features of samples in single cell analysis.

Supplementary Data 8. Related to Figure 6g - h. Summarized information on patient primary tumors and bone marrow sites, including H-scores for phospho-SMAD1/5/9 (marking BMP signaling) in cells co-staining for PHOX2B (marking neuroblastoma cancer cells). Primary tumors in top panel, bone marrow in bottom panel.

Supplementary Data 9. Quantification for western blots.

Supplementary Data 10. Tables for the Methods section, include cell culture conditions, editing construct sequences and screening primers for generation of the cell line with HA-tagged SMAD9, dosing schedule for animal experiments, and the antibodies used for immunofluorescence assays on patient tumor and paired bone marrow samples.
