# Supplementary material for: Bone morphogenetic protein (BMP) signaling determines neuroblastoma cell fate and sensitivity to retinoic acid
Source: Nat Commun. 2025 Feb 28;16:2036. doi: 10.1038/s41467-025-57185-y (PMC11871043; doi:10.1038/s41467-025-57185-y)
Supplement: Supplementary file 13 — Reporting Summary [file 41467_2025_57185_MOESM13_ESM.pdf]

nature portfolio

Corresponding author(s): Min Pan, John Easton, Paul Geeleher

Last updated by author(s): 1/16/2025

Reporting Summary

Nature Portfolio wishes to improve the reproducibility of the work that we publish. This form provides structure for consistency and transparency in reporting. For further information on Nature Portfolio policies, see our Editorial Policies and the Editorial Policy Checklist.

Statistics

For all statistical analyses, confirm that the following items are present in the figure legend, table legend, main text, or Methods section.

n/a	Confirmed	
☐	☒	The exact sample size (*n*) for each experimental group/condition, given as a discrete number and unit of measurement
☐	☒	A statement on whether measurements were taken from distinct samples or whether the same sample was measured repeatedly
☐	☒	The statistical test(s) used AND whether they are one- or two-sided<br>*Only common tests should be described solely by name; describe more complex techniques in the Methods section.*
☒	☐	A description of all covariates tested
☒	☐	A description of any assumptions or corrections, such as tests of normality and adjustment for multiple comparisons
☐	☒	A full description of the statistical parameters including central tendency (e.g. means) or other basic estimates (e.g. regression coefficient) AND variation (e.g. standard deviation) or associated estimates of uncertainty (e.g. confidence intervals)
☐	☒	For null hypothesis testing, the test statistic (e.g. *F*, *t*, *r*) with confidence intervals, effect sizes, degrees of freedom and *P* value noted<br>*Give P values as exact values whenever suitable.*
☒	☐	For Bayesian analysis, information on the choice of priors and Markov chain Monte Carlo settings
☒	☐	For hierarchical and complex designs, identification of the appropriate level for tests and full reporting of outcomes
☒	☐	Estimates of effect sizes (e.g. Cohen's *d*, Pearson's *r*), indicating how they were calculated

Our web collection on statistics for biologists contains articles on many of the points above.

Software and code

Policy information about availability of computer code

Data collection	N/A
Data analysis	Trimgalore v0.4.4, BWA v0.7.17, Samtools v1.9, bedtools v2.17.0, MACS2 v2.1.1, deeptools v3.5.0, DESeq2, clusterProfiler R package, ChIPseeker R package, Pscan-ChIP v1.3, STAR v2.7.10a, RSEM v1.3.3, acNMF (https://rchapple2.github.io/acNMF/)

For manuscripts utilizing custom algorithms or software that are central to the research but not yet described in published literature, software must be made available to editors and reviewers. We strongly encourage code deposition in a community repository (e.g. GitHub). See the Nature Portfolio guidelines for submitting code & software for further information.

Data

Policy information about availability of data

All manuscripts must include a data availability statement. This statement should provide the following information, where applicable:

- Accession codes, unique identifiers, or web links for publicly available datasets
- A description of any restrictions on data availability
- For clinical datasets or third party data, please ensure that the statement adheres to our policy

primary datasets: GSE253785 and GSE253786; referenced datasets GSE216176, GSE137804,EGAD00001008345,GSE228957 and GSE229224

Research involving human participants, their data, or biological material

Policy information about studies with human participants or human data. See also policy information about sex, gender (identity/presentation), and sexual orientation and race, ethnicity and racism.

Reporting on sex and gender	Sex or gender of patients is provided and not considered in this study.
Reporting on race, ethnicity, or other socially relevant groupings	Race or ethnicity of patients is not considered in this study.
Population characteristics	Patient, Primary tumor, Bone marrow, Pre or post treatment, Bone marrow sample collected at:, Retinoic acid treatement prior to sample collection, Status<br>1, S-11-01614, H-13-01918, Post, relapse, no (CAE) for primary tumor YES before bone marrow relapse, alive with recurrent disease<br>2, S-12-01298, H-12-00639, Post, diagnosis, no (CAE), died ~10 years later cause unknown<br>3, S-16-00711, H-16-00303, Pre, diagnosis, no (CAE), alive<br>4, S-16-01131, H-16-00517, Pre, diagnosis, no (cyclo/doxo/melphalan), alive<br>5, S-16-02264, H-16-01267, Pre, post-treatment no (cyclo/topotecan/cisplatin/etoposide/doxo/vincristine), alive<br>6, S-16-02291, H-16-00759, Post, diagnosis, no (per NB2012 induction), died ~6 years later (complications of relapsed neuroblastoma)
Recruitment	Neuroblastoma tissues were obtained from the Tissue Bank at St. Jude Children's Research Hospital.
Ethics oversight	Patient tumor tissues were used under institutional review board (IRB) approval from St. Jude Children's Research Hospital. The patients weren't consented for this study, but all samples were consented for future unspecified research. No participant compensation was provided because this is non-human subjects research so there is no interaction or intervention with the subjects or their identifiable data or biospecimens for this study.

Note that full information on the approval of the study protocol must also be provided in the manuscript.

Field-specific reporting

Please select the one below that is the best fit for your research. If you are not sure, read the appropriate sections before making your selection.

☒ Life sciences ☐ Behavioural & social sciences ☐ Ecological, evolutionary & environmental sciences

For a reference copy of the document with all sections, see nature.com/documents/nr-reporting-summary-flat.pdf

Life sciences study design

All studies must disclose on these points even when the disclosure is negative.

Sample size	Sample sizes were dependent on availability of sensitive neuroblastoma cell line
Data exclusions	No data was excluded from data anlayses
Replication	Replication was performed for high-throughput drug screen and sequencing data
Randomization	No randomization was performed.
Blinding	No blinding was performed

Reporting for specific materials, systems and methods

We require information from authors about some types of materials, experimental systems and methods used in many studies. Here, indicate whether each material, system or method listed is relevant to your study. If you are not sure if a list item applies to your research, read the appropriate section before selecting a response.

Materials & experimental systems

n/a	Involved in the study
	x Antibodies
	x Eukaryotic cell lines
x	Palaeontology and archaeology
	x Animals and other organisms
x	Clinical data
x	Dual use research of concern
x	Plants

Methods

n/a	Involved in the study
	x ChIP-seq
	x Flow cytometry
x	MRI-based neuroimaging

Antibodies

Antibodies used

MAP2 Cell Signaling, # 4542, 1:500 for western blot, 1:200 for IF.
NEFH Cell Signaling, # 55453, 1:200 for IF.
Alexa fluor 594 conjugate anti-rabbit IgG Cell Signaling, # 8889, 1:500.
PHOX2B Abcam, # ab183741, 1:100.
SMAD1/5/9 (phospho S463 + S465 + S467) Abcam, #ab92698, 1:250.
Vimentin Roche, #790-2917, ready to use.
HA tag Cell Signaling, # 3724, 1:50.
SMAD4 R&D Systems, # AF2097, use 20 µg per 20 million cells.
RARA R&D Systems, # PP-H1920-00, use 20 µg per 20 million cells.
β-Actin Sigma, # A1978, 1:20,000.
GAPDH ThermoFisher, # MA5-15738, 1:20,000.
cleaved PARP Cells Signaling, # 5625, 1:1000.
PARP Cell Signaling, # 9542, 1:1000.
SMAD9 ThermoFisher, # 720333, 1:500.
SMAD1 Cell Signaling, # 6944, 1:1000.
pSMAD1/5/9 Cell Signaling, # 13820, 1:500.
Total SMAD1/5/9 Abcam, # ab13723, 1:1000.
ACVR1 Abcam, # ab155981, 1:500.
HRP-linked anti-rabbit IgG Cell Signaling, # 7074, 1:2000.
HRP-linked anti-mouse IgG Cell Signaling, # 7076, 1:2000.
Alexa Fluor 488 conjugated annexin V Thermo Fisher, # A13201, 1:500.

Validation

Antibody Validation
MAP2 Manufacture's website
NEFH Manufacture's website
Alexa fluor 594 conjugate anti-rabbit IgG Manufacture's website
PHOX2B Manufacture's website
SMAD1/5/9 (phospho S463 + S465 + S467) Manufacture's website
Vimentin Manufacture's website
HA tag Manufacture's website
SMAD4 Manuscript
RARA Manuscript
β-Actin Manufacture's website
GAPDH Manufacture's website
cleaved PARP Manufacture's website
SMAD9 Manufacture's website
SMAD1 Manufacture's website
pSMAD1/5/9 Manufacture's website
ACVR1 Manufacture's website
MAP2 Manufacture's website
HRP-linked anti-rabbit IgG Manufacture's website
HRP-linked anti-mouse IgG Manufacture's website
Alexa Fluor 488 conjugated annexin V Manufacture's website

Eukaryotic cell lines

Policy information about cell lines and Sex and Gender in Research

Cell line source(s)

Cell line Source Catlog # Sex
CHP-134 Millipore Sigma, USA 6122002 Male
NB13 St. Jude Children's Research Hospital N/A Male
KELLY Millipore Sigma, USA 92110411 Female

	TGW Xenotech JCRB0618 Male<br>SK-N-SH Millipore Sigma, USA 86012802 Female<br>SK-N-FI ATCC CRL-2142 Male<br>NGP DSMZ (dsmz.de) ACC 676 Male<br>GI-ME-N DSMZ (dsmz.de) ACC 654 Female<br>MHH-NB-11 DSMZ (dsmz.de) ACC 157 Male<br>BE(2)-C ATCC CRL-2268 Male<br>BE(2)-M17 ATCC CRL-2267 Male<br>CHP-212 ATCC CRL-2273 Male<br>SK-N-AS ATCC CRL-2137 Female<br>D283 Med ATCC HTB-185 Male<br>SK-ES-1 ATCC HTB-86 Male<br>SK-MEL-2 ATCC HTB-68 Male<br>HCT 116 ATCC CCL-247 Male<br>RH30 ATCC CRL-2061 Male<br>293T ATCC CRL-3216 Female<br>Cell line Source Catlog # Sex<br>CHP-134 Millipore Sigma, USA 6122002 Male<br>NB13 St. Jude Children's Research Hospital N/A Male<br>KELLY Millipore Sigma, USA 92110411 Female<br>TGW Xenotech JCRB0618 Male<br>SK-N-SH Millipore Sigma, USA 86012802 Female<br>SK-N-FI ATCC CRL-2142 Male<br>NGP DSMZ (dsmz.de) ACC 676 Male<br>GI-ME-N DSMZ (dsmz.de) ACC 654 Female<br>MHH-NB-11 DSMZ (dsmz.de) ACC 157 Male<br>BE(2)-C ATCC CRL-2268 Male<br>BE(2)-M17 ATCC CRL-2267 Male<br>CHP-212 ATCC CRL-2273 Male<br>SK-N-AS ATCC CRL-2137 Female<br>D283 Med ATCC HTB-185 Male<br>SK-ES-1 ATCC HTB-86 Male<br>SK-MEL-2 ATCC HTB-68 Male<br>HCT 116 ATCC CCL-247 Male<br>RH30 ATCC CRL-2061 Male<br>293T ATCC CRL-3216 Female
Authentication	The cell lines were not authenticated.
Mycoplasma contamination	All cell lines were tested negative for mycoplasma using the MycoAlert mycoplasma detection kit (Lonza, LT07-118).
Commonly misidentified lines<br>(See ICLAC register)	No commercially misidentified cell lines were used in this study.

Animals and other research organisms

Policy information about studies involving animals; ARRIVE guidelines recommended for reporting animal research, and Sex and Gender in Research

Laboratory animals	Athymic nude mice (Charles River strain code 553)
Wild animals	No wild animals were used in this study.
Reporting on sex	Animal sex was considered in this study.
Field-collected samples	No field-collected samples were used in this study.
Ethics oversight	The study protocol was approved by the Institutional Animal Care and Use Committee at St. Jude Children's Research Hospital.

Note that full information on the approval of the study protocol must also be provided in the manuscript.

Plants

Seed stocks	No plant used in this study.
Novel plant genotypes	N/A
Authentication	N/A

ChIP-seq

Data deposition

☒ Confirm that both raw and final processed data have been deposited in a public database such as GEO.

☒ Confirm that you have deposited or provided access to graph files (e.g. BED files) for the called peaks.

Data access links<br>*May remain private before publication.*	in the GEO database under the accession numbers GSE253786
Files in database submission	*Provide a list of all files available in the database submission.*
Genome browser session<br>(e.g. UCSC)	*Provide a link to an anonymized genome browser session for "Initial submission" and "Revised version" documents only, to enable peer review. Write "no longer applicable" for "Final submission" documents.*

Methodology

Replicates	Each experiment has two independent replicates.
Sequencing depth	shown in Table S3
Antibodies	HA tag Cell Signaling, # 3724<br>SMAD4 R&D Systems, # AF2097<br>RARA R&D Systems, # PP-H1920-00
Peak calling parameters	MACS2 v2.1.1 was utilized to identify ChIP-seq signal enriched regions (narrow peaks) with the significance cut-off q-value <=0.05
Data quality	identify ChIP-seq signal enriched regions with the significance cut-off q-value <=0.05
Software	trimgalore v0.4.4 was applied to remove low quality reads and adaptor with default setting<br>ChIP-seq reads were aligned to the UCSC hg38 genome using BWA mem v0.7.17 with default setting<br>To generate genome-wide coverage profiles, the bedtools v2.17.0 “genomecov” command was applied , and the output was converted to bigwig files using bedGraphToBigWig<br>MACS2 v2.1.1 was utilized to identify ChIP-seq signal enriched regions (narrow peaks) with the significance cut-off q-value <=0.05

Flow Cytometry

Plots

Confirm that:

☒ The axis labels state the marker and fluorochrome used (e.g. CD4-FITC).

☒ The axis scales are clearly visible. Include numbers along axes only for bottom left plot of group (a 'group' is an analysis of identical markers).

☒ All plots are contour plots with outliers or pseudocolor plots.

☒ A numerical value for number of cells or percentage (with statistics) is provided.

Methodology

Sample preparation	For apoptosis, cells were treated in 6-well plates. After incubation for the time needed, cell culture medium and cells were collected and washed in cold phosphate-buffered saline (PBS). The cells were then resuspended in annexin-binding buffer and stained with Alexa Fluor 488 conjugated annexin V (Thermo Fisher, catalog# A13201) and 1 µg/ml Propidium iodide (PI) for flow cytometry analysis.

Instrument	BD LSRFortess X-20
Software	BD FACSDiva and FlowJo
Cell population abundance	10,000 events were collected for each replicate. Single cells were used for analysis. Single cells were defined by cell size using FSC-A and SSC-W. Details are included in Supplementary figure 11.
Gating strategy	Detailed gating strategies are included in Supplementary figure 11.

☒ Tick this box to confirm that a figure exemplifying the gating strategy is provided in the Supplementary Information.
